# An inflammatory liquid fingerprint predicting tumor recurrence after liver transplantation for hepatocellular carcinoma

**DOI:** 10.1002/mco2.678

**Published:** 2024-08-26

**Authors:** Modan Yang, Zuyuan Lin, Li Zhuang, Linhui Pan, Rui Wang, Hao Chen, Zhihang Hu, Wei Shen, Jianyong Zhuo, Xinyu Yang, Huigang Li, Chiyu He, Zhe Yang, Qinfen Xie, Siyi Dong, Junli Chen, Renyi Su, Xuyong Wei, Junjie Yin, Shusen Zheng, Di Lu, Xiao Xu

**Affiliations:** ^1^ Department of Breast Surgery The Second Affiliated Hospital Zhejiang University School of Medicine Hangzhou China; ^2^ NHC Key Laboratory of Combined Multi‐Organ Transplantation Zhejiang University Hangzhou China; ^3^ Key Laboratory of Integrated Oncology and Intelligent Medicine of Zhejiang Province Affiliated Hangzhou First People's Hospital, School of Medicine, Westlake University Hangzhou China; ^4^ Zhejiang University School of Medicine Hangzhou China; ^5^ Department of Hepatobiliary and Pancreatic Surgery Shulan (Hangzhou) Hospital Hangzhou China; ^6^ Department of Hepatobiliary and Pancreatic Surgery Affiliated Hangzhou First People's Hospital School of Medicine Westlake University Hangzhou China; ^7^ National Center for Healthcare Quality Management in Liver Transplant Hangzhou China; ^8^ Department of Hepatobiliary & Pancreatic Surgery and Minimally Invasive Surgery Zhejiang Provincial People's Hospital (Affiliated People's Hospital) School of Clinical Medicine Hangzhou Medical College Hangzhou China; ^9^ Institute of Translational Medicine Zhejiang University School of Medicine Hangzhou China

**Keywords:** cytokines, hepatocellular carcinoma, liver transplantation, prognostic model, T‐cell profiling

## Abstract

Tumor recurrence is a life‐threatening complication after liver transplantation (LT) for hepatocellular carcinoma (HCC). Precise recurrence risk stratification before transplantation is essential for the management of recipients. Here, we aimed to establish an inflammation‐related prediction model for posttransplant HCC recurrence based on pretransplant peripheral cytokine profiling. Two hundred and ninety‐three patients who underwent LT in two independent medical centers were enrolled, and their pretransplant plasma samples were sent for cytokine profiling. We identified four independent risk factors, including alpha‐fetoprotein, systemic immune‐inflammation index, interleukin 6, and osteocalcin in the training cohort (*n* = 190) by COX regression analysis. A prediction model named inflammatory fingerprint (IFP) was established based on the above factors. The IFP effectively predicted posttransplant recurrence (area under the receiver operating characteristic curve [AUROC]: 0.792, C‐index: 0.736). The high IFP group recipients had significantly worse 3‐year recurrence‐free survival rates (37.9 vs. 86.9%, *p* < 0.001). Simultaneous T‐cell profiling revealed that recipients with high IFP were characterized by impaired T cell function. The IFP also performed well in the validation cohort (*n* = 103, AUROC: 0.807, C‐index: 0.681). In conclusion, the IFP efficiently predicted posttransplant HCC recurrence and helped to refine pretransplant risk stratification. Impaired T cell function might be the intrinsic mechanism for the high recurrence risk of recipients in the high IFP group.

## Graphical abstract

• Conjoint analysis of pretransplant peripheral inflammatory and T‐cell profiling depicts recurrence‐related immune status.

• The inflammatory fingerprint (IFP) incorporates interleukin 6, osteocalcin, systemic immune‐inflammation index, and α‐fetoprotein to predict recurrence after LT for HCC and significantly optimizes the current risk stratification system.

• Impaired T cell function could primly elucidate why the IFP can precisely predict tumor recurrence after LT for HCC.

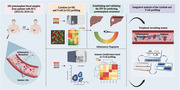

## 1 INTRODUCTION

Hepatocellular carcinoma (HCC) is one of the most common malignancies and ranks the highest mortality among cancers in Chinese males under 60 years old.^1^, ^2^, ^3^ Liver transplantation (LT) is currently the most effective intervention that removes both the tumor and the pathogenic liver, leading to a favorable prognosis in patients with HCC who meet the candidate selection criteria.

In China, HCC‐related LT accounts for approximately 35.0% of LT cases, according to the China Liver Transplant Registry (CLTR) reports (2020),^4^ and this rate is much lower in the Western world, 12.6% in the United States in 2020,^5^ for instance. Posttransplant recurrence is still a dominant threat to the long‐term survival of recipients. Despite carefully selecting recipients based on different criteria, the 5‐year recurrence rate of HCC following LT is still as high as 30%.^6^ Therefore, it is essential to optimize the risk stratification system to guide the individualized prevention and intervention strategies for recipients to ensure the therapeutic efficacy and sufficient utilization of scarce organ resources.^7^

Currently, the candidate selection criteria for HCC‐related LT are often applied to evaluate posttransplant recurrence risk. For instance, the Milan criteria are the most classic and strict candidate selection criteria, and the University of San Francisco California (UCSF) criteria and Tokyo criteria are also commonly used in clinical practice.^8^, ^9^, ^10^ Hangzhou criteria proposed by Zheng et al.^11^ have made the candidate selection more reasonable by incorporating tumor biological biomarker α‐fetoprotein (AFP) and pathological classification. A few years ago, Xu et al.^7^ proposed a brand‐new risk stratification system based on the Hangzhou criteria and showed good application values in the Chinese population. However, recipients who fulfill the criteria may still suffer from tumor recurrence after LT, while many recipients could achieve long‐term recurrence‐free survival (RFS) even if they exceed the criteria, suggesting that an accurate and reliable prediction model is still a promising research direction for precise diagnosis and treatment for patients with HCC.

As HCC is typically an inflammation‐related malignancy, the interactions between tumor biological behavior and host immune response are inextricably related to posttransplant recurrence. Previous studies have reported that inflammation‐associated hematologic parameters, such as systemic immune‐inflammation index (SII), neutrophil‐to‐lymphocyte ratio (NLR), platelet‐to‐lymphocyte ratio (PLR), are promising prognostic indicators for HCC recurrence after LT.^12^ Notably, cytokine profiling provides a new perspective for prognostic biomarker exploration. For instance, the tumor necrosis factor (TNF) superfamily proteins,^13^, ^14^, ^15^ interferon (IFN) family proteins, and T regulator (Treg) cytokines^16^, ^17^, ^18^ released in the process of inflammatory‐immunological interactions are useful and noninvasive signals for tracing tumor recurrence.^19^, ^20^, ^21^ T cells are the core force of antitumor immunity, dominate graft immune rejection, and are important regulators of inflammatory immune homeostasis in LT recipients with HCC.^22^ Mature T cells (CD3^+^) can be broadly divided into CD4^+^, CD8^−^ and CD4^−^, CD8^+^ subsets. CD4^+^ T cells mainly play an auxiliary function and regulate the response of other immune cells. CD8^+^ T cells, on the other hand, are cytotoxic and responsive “guardians” that can quickly eliminate invading pathogens and tumor cells. Research suggests that postoperative tumor recurrence is often accompanied by abnormal expression of regulatory T cells and related cytokines in peripheral blood.^23^, ^24^ Therefore, the immune classification based on T cell composition and functional changes is conducive to assessing the antitumor immune status of recipients, which might provide in‐depth insights into pretransplant risk stratification.

**FIGURE 1:**
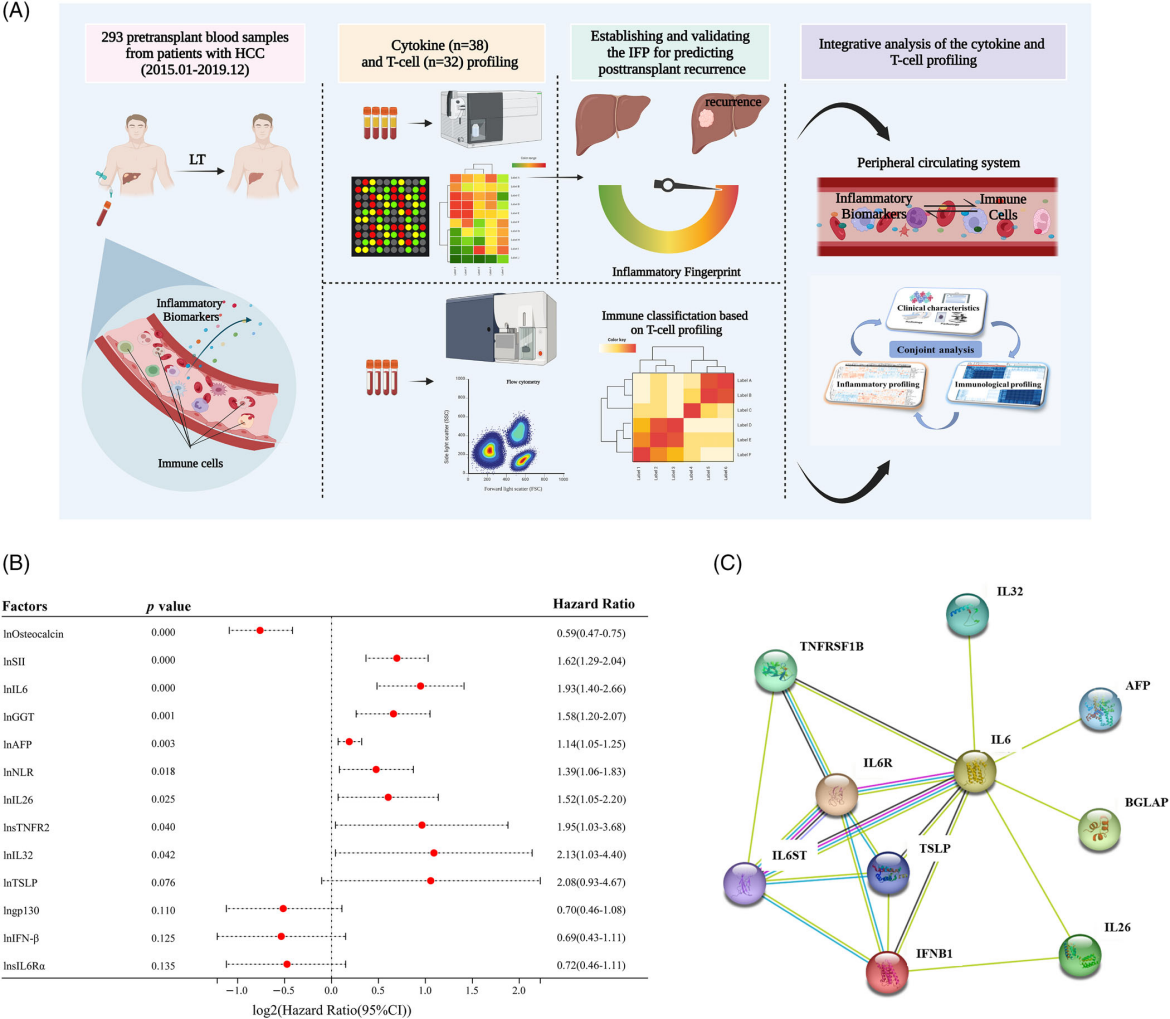
The schematic diagram of research, forest plot, and PPI network of recurrence‐related factors. (A) The schematic diagram of current research. (B) Forest plot of recurrence‐related factors. Thirteen factors were identified and presented by the forest plot. (C) PPI network of recurrence‐related biomarkers (*n* = 10). IL6 was the core molecule of the network. BGLAP, osteocalcin; IL6ST, interleukin 6 receptor subunit beta (gp130); IFNB1, interferon beta one; TNFRSF1B, tumor necrosis factor receptor superfamily member 1B, TNFR2; PPI, protein–protein interaction.

In this study, we aimed to establish a prediction model for posttransplant HCC recurrence based on pretransplant peripheral cytokine profiling and try to provide elucidation of the model's predictive capacity through T‐cell profiling and interpretation. Figure 1A shows a schematic illustration of our research.

## 2 RESULTS

### 2.1 Baseline demographic and clinical characteristics of the studied population

A total of 293 patients with HCC who underwent LT (hereafter referred to as recipients) and had complete clinical information and available pretransplant blood samples from two independent transplant centers were enrolled in this study. The median follow‐up time in the training and validation cohorts was 3.0 and 3.9 years, respectively. The demographic and clinical characteristics of recipients in the training and validation cohorts are shown in Table 1. Both the training (14 females and 176 males) and validation cohorts (11 females and 92 males, *p* = 0.333) had more than 60% of patients who were over 50 years old (*p* = 0.700). A total of 96.8 and 98.1% of recipients in the training and validation cohorts had liver cirrhosis (*p* = 0.542), and hepatitis B virus (HBV) infection rates were 86.8 and 91.3% (*p* = 0.259), respectively. Recipients with model for end‐stage liver disease (MELD) scores over 26 accounted for 57.4 and 8.7% in the training and validation cohorts (*p *< 0.001). The tumor features did not differ between the training and validation cohorts: 47.4 versus 53.4% recipients had multifocal lesions (*p* = 0.324), 25.3 versus 31.1% recipients had the max tumor lesion larger than 5 cm (*p* = 0.287), 26.8 versus 35.0% recipients had the total tumor lesion over 10 cm (*p* = 0.147). 53.7 and 60.2% of recipients received pretransplant down‐stage treatment in the training and validation cohort (*p* = 0.284). The pretransplant AFP, SII, alanine aminotransferase (ALT), and aspartate aminotransferase (AST) were consistent between the training and validation cohorts, as shown in Table 1. At the same time, the NLR and γ‐glutamyltranspeptidase (GGT) were different between the two cohorts. There were 45 and 54 recipients who suffered HCC relapse after LT in the training and validation cohort, respectively.

**TABLE 1:** Demographic and clinical characteristics of patients in different cohorts.

	Whole population	Training cohort	Validation cohort	
Characteristics	Number of cases (%)^a^	Number of cases (%)	Number of cases (%)	*p* Value^*^
Total	293	190 (64.8)	103 (35.2)	
Age (year)				0.700
≤50	101 (34.5%)	64 (33.7%)	37 (35.9%)	
>50	192 (65.5%)	126 (66.3%)	66 (64.1%)	
Gender				0.333
Male	268 (91.5%)	176 (92.6%)	92 (89.3%)	
Female	25 (8.5%)	14 (7.4%)	11 (10.7%)	
BMI				0.886
≤25	212 (72.4%)	138 (72.6%)	74 (71.8%)	
>25	81 (27.6%)	52 (27.4%)	29 (28.2%)	
Hepatitis B virus infection				0.259
No	34 (11.6%)	25 (13.2%)	9 (8.7%)	
Yes	259 (88.4%)	165 (86.8%)	94 (91.3%)	
Cirrhosis				0.542
No	8 (2.7%)	6 (3.2%)	2 (1.9%)	
Yes	285 (97.3%)	184 (96.8%)	101 (98.1%)	
MELD score				<0.001
≤26	175 (59.7%)	81 (42.6%)	94 (91.3%)	
>26	118 (40.3%)	109 (57.4%)	9 (8.7%)	
DS/LR				0.284
No	129 (44.0%)	88 (46.3%)	41 (39.8%)	
Yes	164 (56.0%)	102 (53.7%)	62 (60.2%)	
Tumor number				0.324
Single	148 (50.5%)	100 (52.6%)	48 (46.6%)	
Multifocal	145 (49.5%)	90 (47.4%)	55 (53.4%)	
Max tumor diameter (cm)				0.287
≤5	213 (72.7%)	142 (74.7%)	71 (68.9%)	
>5	80 (27.3%)	48 (25.3%)	32 (31.1%)	
Total tumor diameter (cm)				0.147
≤10	206 (70.3%)	139 (73.2%)	67 (65.0%)	
>10	87 (29.7%)	51 (26.8%)	36 (35.0%)	
IL6 (pg/mL)^b^	13.4 (11.1–16.0)	13.4 (10.3–14.5)	13.7 (11.9–18.6)	0.800
AFP (ng/mL)	19 (4.5–161.2)	18.8 (49.1–132.1)	20.4 (4.6–180.2)	0.790
ALT (U/L)	35.0 (24.0–51.0)	35.0 (24.0–49.3)	35.0 (24.0–54.0)	0.890
AST (U/L)	42.0 (32.0–66.5)	42.0 (28.0–65.5)	42.0 (34.0–67.0)	0.195
GGT (U/L)	73.0 (39.0–124.5)	64.5 (33.8–113.5)	91.0 (57.0–149.0)	0.001
NLR	3.3 (2.2–5.2)	3.4 (2.3–5.5)	3.2 (2.0–4.3)	0.017
SII	262.4 (148.6–457.8)	262.0 (145.6–500.6)	262.5 (150.8–358.7)	0.318
Milan criteria				0.027
Fulfilling	148 (50.5%)	105 (55.3%)	43 (41.7%)	
Exceeding	145 (49.5%)	85 (44.7%)	60 (58.3%)	
UCSF criteria				0.067
Fulfilling	180 (61.4%)	124 (65.3%)	56 (54.4%)	
Exceeding	113 (38.6%)	66 (34.7%)	47 (45.6%)	
Tokyo criteria				0.115
Fulfilling	180 (61.4%)	123 (64.7%)	57 (55.3%)	
Exceeding	113 (38.6%)	67 (35.3%)	46 (44.7%)	

^a^ Categorical variables are expressed as the count (*n*) and proportion (percentage).

^b^ Non‐normally distributed continuous variables were presented as medians (IQR, interquartile range).

* Student's *t*‐test was utilized to analyze normally distributed continuous variables, and the Mann–Whitney test or Wilcoxon rank sum test was used for non‐normally distributed continuous variables, Pearson's chi‐square test or Fisher's exact test was applied to analyze categorical variables.

### 2.2 Recurrence‐related inflammatory biomarkers and clinical variables

Thirty‐eight cytokines and six commonly used clinical systemic inflammatory indexes and tumoral biomarkers, including ALT, AST, GGT, SII, NLR and AFP, were detected for the whole study population. Before the COX regression analysis, we tested the proportional hazards (PH) assumption for a Cox regression model fit. The results showed that all the forty‐four variables fitted the PHs assumption (*p* > 0.050; Figure S1). The above results supported our further conducting Cox proportional hazards regression analysis to identify independent risk factors for posttransplant recurrence. We applied univariate Cox proportional hazards regression analysis to screen out recurrence‐related inflammatory biomarkers, and the results are shown in Table 2. To enroll all the recurrence‐related biomarkers, variables with a *p* value less than 0.15 were selected for subsequent analysis. Thirteen variables were identified as recurrence‐related factors including lnAFP (Hazard Rario [HR]: 1.14, 1.05–1.25; *p* = 0.003), lnNLR (HR: 1.39, 1.06–1.83; *p* = 0.018), lnSII (HR: 1.62, 1.29–2.04; *p* < 0.001), lnGGT (HR: 1.58, 1.20–2.07; *p* = 0.001), lngp130 (HR: 0.70, 0.46–1.08; *p* = 0.110), lnIFN‐β (HR: 0.69, 0.43–1.11; *p* = 0.125), lnsIL6Rα (HR: 0.72, 0.46–1.11; *p* = 0.135), lnIL26 (HR: 1.52, 1.05–2.20; *p* = 0.025), lnIL32 (HR: 2.13, 1.03–4.40; *p* = 0.042), lnOsteocalcin (HR: 0.59, 0.47–0.75; *p* < 0.001), lnsTNFR2 (HR: 1.95, 1.03–3.68; *p* = 0.040), lnTSLP (HR: 2.08, 0.93–4.67; *p* = 0.076), lnIL6 (HR: 1.93, 1.40–2.66; *p* < 0.001). We established a forest plot to present these results (Figure 1B). To gain deeper insight into those recurrence‐related biomarkers (*n* = 10), we mapped the protein–protein interaction network based on the String database. We found that IL6 was the core molecule of the network (Figure 1C), which was in line with our previous findings (unpublished data).

**TABLE 2:** Cox regression analysis of peripheral inflammatory and tumoral factors (*n* = 44) in the training cohort.

	Univariate analysis			Multivariate analysis		
Variables	HR	95%CI	*p* Value^*^	HR	95%CI	*p* Value
APRIL^a^	0.86	0.58–1.27	0.438			
BAFF	1.11	0.73–1.69	0.614			
sCD30	1.00	0.66–1.54	0.983			
sCD163	0.83	0.57–1.20	0.318			
Chitinase3‐like1	0.74	0.45–1.20	0.215			
**gp130/sIL‐6Rβ**	**0.70**	**0.46–1.08**	**0.110**			0.724
IFN‐α2	1.14	0.54–2.40	0.723			
**IFN‐β**	**0.69**	**0.43–1.11**	**0.125**			0.162
IFN‐γ	0.87	0.51–1.50	0.627			
IL2	1.17	0.81–1.69	0.393			
**sIL6Ra**	**0.72**	**0.46–1.11**	**0.135**			0.756
IL8	1.04	0.87–1.24	0.686			
IL10	1.52	0.86–2.70	0.152			
IL11	1.55	0.76–3.17	0.231			
IL12‐p40	1.36	0.74–2.51	0.326			
IL12‐p70	0.96	0.83–1.11	0.567			
IL19	0.81	0.59–1.11	0.186			
IL20	1.29	0.54–3.07	0.572			
IL22	1.16	0.89–1.52	0.261			
**IL26**	**1.52**	**1.05–2.20**	**0.025**			0.299
IL27	0.95	0.72–1.24	0.692			
IL28A	1.26	0.59–2.72	0.551			
IL29	1.34	0.76–2.35	0.315			
**IL32**	**2.13**	**1.03–4.40**	**0.042**			0.467
IL34	1.08	0.75–1.55	0.680			
IL35	1.06	0.68–1.64	0.797			
LIGHT	1.05	0.88–1.26	0.560			
MMP‐1	1.17	0.68–1.98	0.573			
MMP‐2	0.80	0.58–1.09	0.161			
MMP‐3	0.78	0.52–1.18	0.245			
**Osteocalcin**	**0.59**	**0.47–0.75**	**<0.001**	**0.68**	**0.51–0.90**	**0.013**
Osteopontin	1.15	0.65–2.01	0.633			
Pentraxin‐3	1.02	0.76–1.38	0.877			
sTNF‐R1	1.13	0.72–1.78	0.599			
**sTNF‐R2**	**1.95**	**1.03–3.68**	**0.040**			0.334
**TSLP**	**2.08**	**0.93–4.67**	**0.076**			0.827
TWEAK	1.00	0.76–1.32	0.983			
**IL6**	**1.93**	**1.40–2.66**	**<0.001**	**2.17**	**1.51–3.11**	**<0.001**
**NLR**	**1.39**	**1.06–1.83**	**0.018**			0.115
**SII**	**1.62**	**1.29–2.04**	**<0.001**	**1.40**	**1.08–1.81**	**0.009**
ALT	1.10	0.85–1.41	0.469			
AST	1.13	0.90–1.41	0.293			
**GGT**	**1.58**	**1.20–2.07**	**0.001**			0.346
**AFP**	**1.14**	**1.05–1.25**	**0.003**	**1.14**	**1.04–1.24**	**0.005**

Abbreviations: APRIL, a proliferating‐inducing ligand; BAFF, B‐cell activating factor; IFN‐α2, interferon α2; IFN‐β, interferon β; IFN‐γ, interferon γ; IL2, interleukin 2; sIL6Rα, soluble interleukin 6 receptor α; IL8, interleukin 8; IL10, interleukin 10; IL11, interleukin 11; IL12‐p40, interleukin 12(p40); IL12‐p70, interleukin 12(p70); IL19, interleukin 19; IL20, interleukin 20; IL22, interleukin 22; IL26, interleukin 26; IL27, interleukin 27; IL28A, interleukin 28A; IL29, interleukin 29; IL32, interleukin 32; IL34, interleukin 34; IL35, interleukin 35; LIGHT, TNFRSF14; MMP, matrix metalloproteinase; TSLP, thymic stromal lymphopoietin; sTNF‐R1, soluble tumor necrosis factor receptor 1; sTNF‐R2, soluble tumor necrosis factor receptor 2; TWEAK, TNF‐related weak inducer of apoptosis; CI, confidence interval.

^a^ All concentration data were natural log‐transformed and standardized. The natural logarithm symbol “ln” was omitted in the table for space‐saving and aesthetic reasons.

* The univariate and multivariate Cox proportional hazards regression analysis were used to identify recurrence‐associated variables and independent prognostic factors.

### 2.3 Inflammatory fingerprint incorporated four biomarkers and predicted HCC recurrence after LT in the training cohort

Multivariate Cox PHs regression analysis identified four independent risk factors including lnAFP (HR: 1.14, 1.04–1.24; *p* = 0.005), lnSII (HR: 1.40, 1.08–1.81; *p* = 0.009), lnIL6 (HR: 2.17, 1.51–3.11; *p* < 0.001), lnOsteocalcin (HR: 0.68, 0.51–0.90; *p* = 0.013) (Table 2). Their area under the receiver operating characteristic curve (AUROC) was 0.600, 0.686, 0.617, and 0.704, respectively (Figure S2A–D). The Kaplan–Meier (KM) plot presented the survival analysis results of the four inflammatory biomarkers based on the optimal cut‐off values (Figure S2E–H). The optimal cut‐off values of lnAFP, lnSII, lnIL6, and lnOsteocalcin were 5.7, 5.5, 2.5, and 7.4, respectively. It should be pointed out that all four biomarkers had stable expression levels in the training and validation cohorts (Figure S3A–D).

**FIGURE 2:**
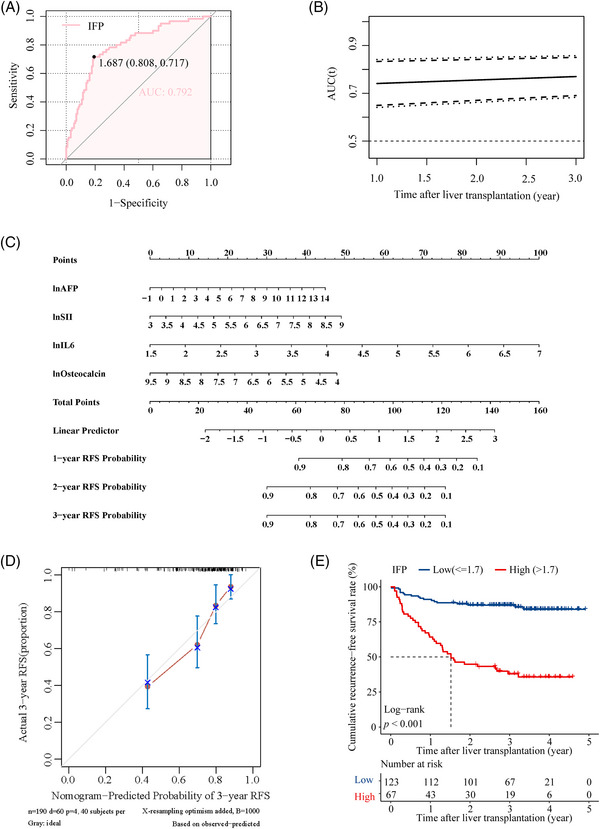
The IFP predicts posttransplant HCC recurrence in the training cohort. (A) ROC curve for the IFP in the training cohort. The AUROC was 0.792. (B) Time‐dependent ROC curve for the IFP in the training cohort. The AUROC increased over time. (C) The nomogram of the IFP in the training cohort. (D) Calibration curve of the IFP in predicting 3‐year RFS in the training cohort. (E) RFS curve for the IFP in the training cohort. Recipients in the low IFP and high IFP groups had significantly different RFS (*p* < 0.001). The 3‐year RFS rate was 86.9 and 37.9%, respectively.

A digital prognostic model named inflammatory fingerprint (IFP) was developed based on the coefficient value of the inflammatory biomarkers, and the value of the IFP was calculated using the formula: 0.76 × lnIL6 + 0.35 × lnSII + 0.13 × lnAFP − 0.37 × lnOsteocalcin. The optimal cut‐off value of IFP determined by receiver operating characteristic (ROC) analysis was 1.7, and the AUROC was 0.792 (Figure 2A); the time‐dependent ROC curve showed that the AUROC of the IFP increased over time (Figure 2B); the 1, 2, and 3‐year AUROC was demonstrated in Figure S3E separately. Then, we established a predictive nomogram based on the Cox PHs regression analysis (Figure 2C). The overall index of concordance (C‐index) was 0.736, indicating satisfactory predictive discrimination of the IFP. Figure 2D showed the calibration curve of the IFP in predicting 3‐year RFS in the training cohort, and the Brier score was 0.178 (95% CI, 0.147–0.209) (Figure S4A), indicating good predictive accuracy of the IFP. Subsequently, we divided recipients in the training cohort into the high IFP group (*n* = 67) and the low IFP group (*n* = 123). KM analysis indicated that recipients in the high IFP group and low IFP group had a significant difference in RFS (HR: 7.43, 95% CI, 4.28–12.92, *p* < 0.001; Figure 2E); the 3‐year RFS rate was 37.9 and 86.9%, respectively. The results were identical in the analysis using a competing‐risk model (Figure S4C). According to our study, the traditional candidate selection criteria, including the Milan criteria, UCSF criteria, and Tokyo criteria, performed well in predicting posttransplant HCC recurrence for recipients in the training cohort. Recipients who fulfilled the criteria had significantly better RFS than those who exceeded the criteria (*p* < 0.050; Figure S5A,C,E). The 3‐year RFS rate was 81.8 versus 53.8%, 77.3 versus 54.1%, and 81.2 versus 47.4% for patients who fulfilled or exceeded the three criteria (Milan criteria, UCSF criteria, and Tokyo criteria), respectively. To further explore the superiority of the IFP, we compared it with the three traditional criteria through the net reclassification improvement (NRI) and the integrated discrimination index (IDI). The NRI was 27.0, 27.0, and 25.3% compared with the Milan, UCSF, and Tokyo criteria. The IDI also supported the superiority of the IFP as it was 10.3, 10.3, and 10.2% when compared with the Milan, UCSF, and Tokyo criteria, respectively (Figure S5B,D,F). The NRI and IDI indicated that the IFP had better predictive capacity in predicting posttransplant HCC recurrence in the training cohort.

**FIGURE 3:**
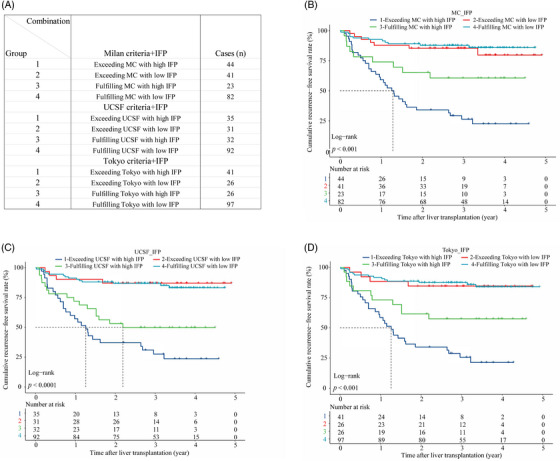
The combination of the IFP and traditional selection criteria predicts posttransplant HCC recurrence in the training cohort. (A) The combination of the IFP and the traditional candidate selection criteria and subgrouping arrangement. (B) RFS curve for the subgroups of the IFP and Milan criteria combination in the training cohort. (C) RFS curve for the subgroups of the IFP and the UCSF criteria combination in the training cohort. (D) RFS curve for the subgroups of the IFP and Tokyo criteria combination in the training cohort.

Combined analysis showed that the IFP could optimize the recurrence risk stratification based on the traditional criteria in the training cohort. We created new subgroups by combining the criteria with the IFP as follows (Figure 3A): (1) “MC+IFP” ① Group 1: exceeding MC with high IFP (*n* = 44); ② Group 2: exceeding MC with low IFP (*n* = 41); ③ Group 3: fulfilling MC with high IFP (*n* = 23); ④ Group 4: fulfilling MC with low IFP (*n* = 82). (2) “UCSF+IFP” ① Group 1: exceeding UCSF with high IFP (*n* = 35); ② Group 2: exceeding UCSF with low IFP (*n* = 31); ③ Group 3: fulfilling UCSF with high IFP (*n* = 32); ④ Group 4: fulfilling UCSF with low IFP (*n* = 92). (3) “Tokyo+IFP” ① Group 1: exceeding Tokyo with high IFP (*n* = 41); ② Group 2: exceeding Tokyo with low IFP (*n* = 26); ③ Group 3: fulfilling Tokyo with high IFP (*n* = 26); ④ Group 4: fulfilling Tokyo with low IFP (*n* = 97). First of all, we found that nearly half of recipients who exceeded the criteria had low IFP and better RFS, which was close to that of recipients fulfilling the criteria with low IFP. Specifically, recipients in groups 2 and 4 of the “MC+IFP” combination had similar RFS (*p* > 0.050) and the 3‐year RFS rates were 85.4 versus 87.7%, respectively. And recipients in groups 2 and 4 of the “UCSF+IFP” combination also had similar RFS (*p* > 0.050), and the 3‐year RFS rates were 87.1 versus 86.9%, respectively. Likewise, recipients in groups 2 and 4 of the “Tokyo+IFP” combination had similar RFS (*p* > 0.050), and the 3‐year RFS rates were 84.6 versus 87.6%, respectively. To our delight, the IFP could also screen out recipients with high recurrence risk even if they met the criteria. The KM analysis showed that recipients who fulfilled the criteria with a high IFP had poorer RFS, which was close to that of recipients who exceeded the criteria with a high IFP. For instance, recipients in groups 1 and 3 of the “MC+IFP” combination had worse RFS, and the 3‐year RFS rates were 26.3 and 60.6%, respectively. And recipients in groups 1 and 3 of the “UCSF+IFP” combination also had worse RFS, and the 3‐year RFS rates were 27.5 and 49.8%, respectively. Likewise, recipients in groups 1 and 3 of the “Tokyo+IFP” combination had worse RFS, and the 3‐year RFS rates were 25.7 and 57.4% (Figure 3B–D). These results indicated that the IFP could refine the recurrence risk stratification based on the traditional candidate selection criteria.

Additionally, we found that the IFP also performed well in predicting posttransplant overall survival (OS) of patients in the training cohort, with AUROC at 0.761 (Figure S6A). Recipients in the low IFP group had better OS than recipients in the high IFP group (HR: 6.40, 95% CI: 3.50–11.68, *p* < 0.001; Figure S6B); the 3‐year OS rate was 87.9 and 53.8%, respectively.

### 2.4 Recipients in the high IFP group were characterized by impaired peripheral T cell function

**FIGURE 4:**
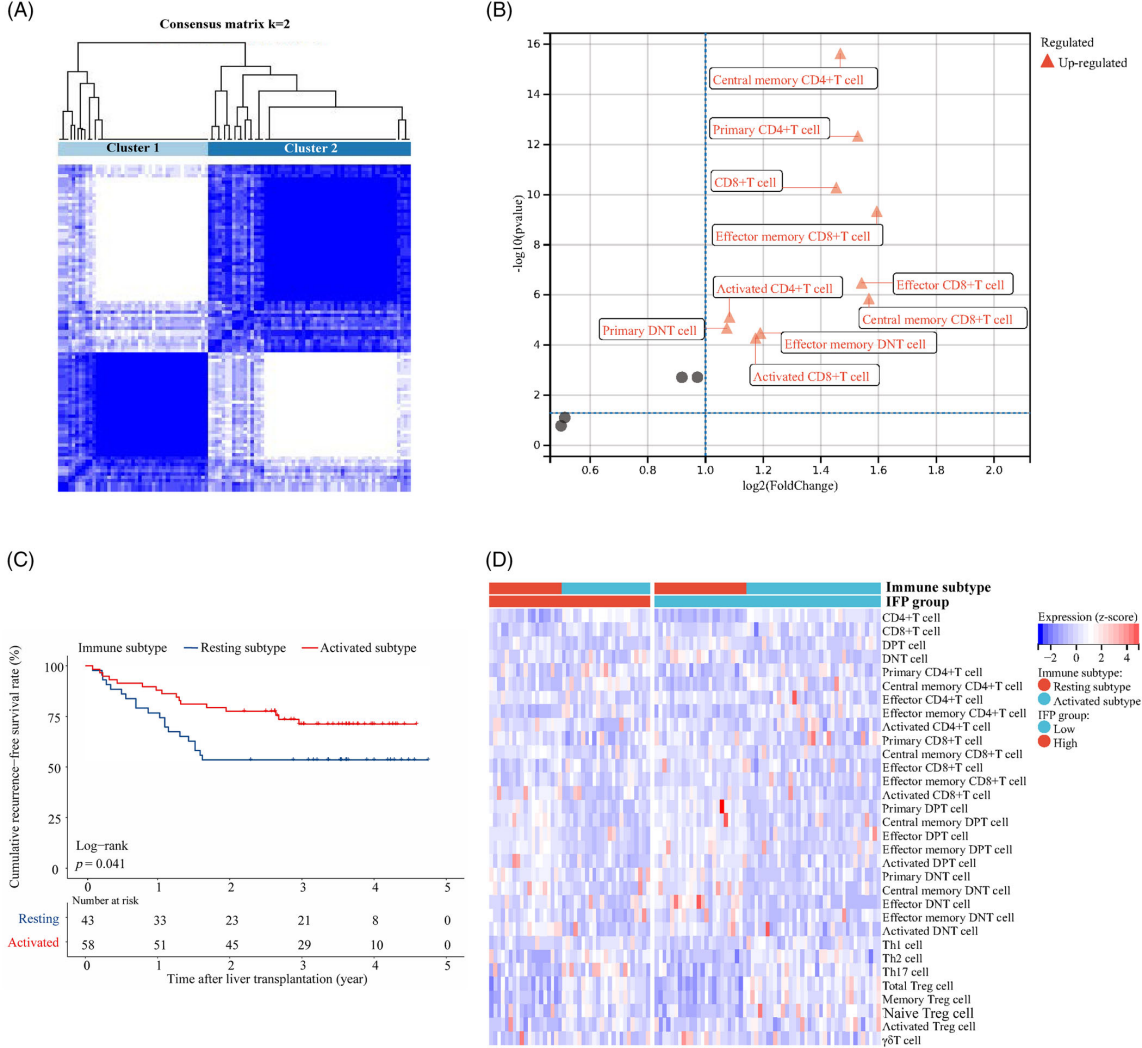
Exploration of the immune classification of recipients (*n* = 101) T‐cell profiling (*n* = 32). (A) Heat map of consistency cluster analysis (cluster 1: *n* = 43; cluster 2: *n* = 58). (B) Volcano plot of the differentially expressed T cell subsets in cluster 1 and cluster 2 (cluster 2 vs. cluster 1). (C) RFS curve for the immune classification. Recipients in the activated and resting classifications had significantly different RFS (*p* = 0.041). The 3‐year RFS rate was 71.3 and 53.5%, respectively. (D) Heat map of T cell subsets annotated with the IFP and the immune classification.

We carried out flow cytometry (FC) to profile the peripheral T cell status in 101 out of the 190 studied recipients in the training cohort (Table S1). Thirty‐two subsets of peripheral blood T cells were monitored, and their absolute expression levels (count/µL) were used for further analysis. The consensus cluster analysis identified two clusters characterized by different T cell function statuses (cluster 1: *n* = 43, cluster 2: *n* = 58; Figure 4A). Recipients in cluster 2 had higher expression levels of ten T cell subsets, including total CD8^+^ T (log2FC = 1.45, *p* < 0.001), activated CD8^+^ T (log2FC = 1.17, *p* < 0.001), effector CD8^+^ T (log2FC = 1.54, *p* < 0.001), effector memory CD8^+^ T (log2FC = 1.59, *p* < 0.001), central memory CD8^+^ T (log2FC = 1.57, *p* < 0.001), activated CD4^+^ T (log2FC = 1.09, *p* < 0.001), central memory CD4^+^ T (log2FC = 1.47, *p* < 0.001), primary CD4^+^ T (log2FC = 1.53, *p* < 0.001), effector memory double negative T (DNT) (log2FC = 1.19, *p* < 0.001), and primary DNTs (log2FC = 1.07, *p* < 0.001) than recipients in cluster 1. The results were shown by volcano plot (Figure 4B). As most of the above T cell subsets had activated functional status, we named cluster 1 the “resting” classification and cluster 2 the “activated” classification. Recipients in the activated classification had significantly better RFS than those recipients in the resting classification (HR: 1.96, 95% CI: 1.01–3.85, *p* = 0.041; Figure 4C). The 3‐year RFS rate was 71.3 and 53.5%, respectively. Further analysis found that recipients with high IFP were mainly in the resting classification and provided a primary explanation for the predictive capacity of the IFP (Figure 4D).

### 2.5 External validation of the IFP in predicting HCC recurrence after LT

**FIGURE 5:**
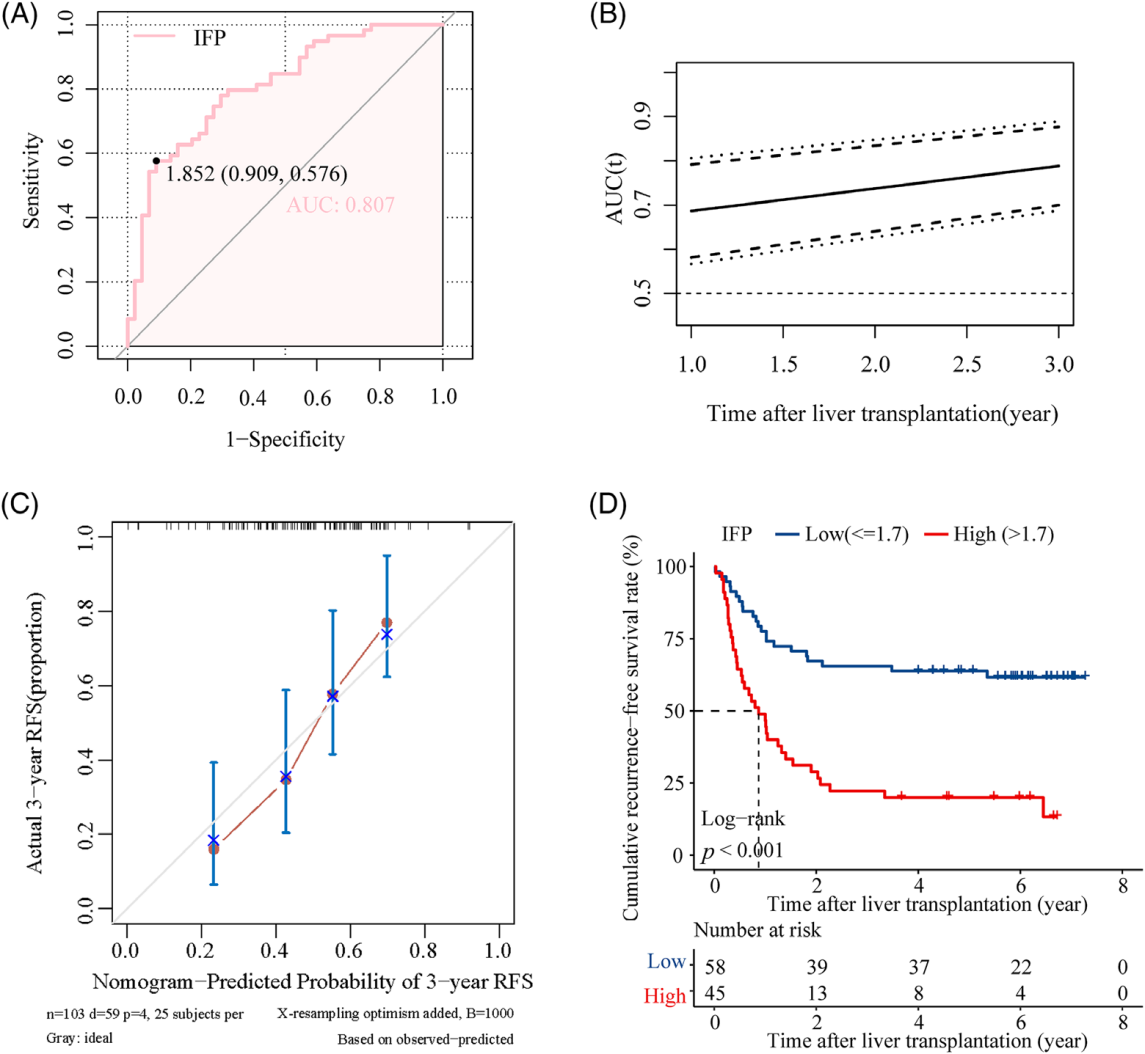
The IFP predicts posttransplant HCC recurrence in the validation cohort. (A) ROC curve for the IFP in the validation cohort. The AUROC was 0.807. (B) Time‐dependent ROC curve for the IFP in the validation cohort. The AUROC increased over time. (C) Calibration curve of the IFP in predicting 3‐year RFS in the validation cohort. (D) RFS curve for the IFP in the validation cohort. Recipients in the low IFP and high IFP groups had significantly different RFS (*p* < 0.001). The 3‐year RFS rate was 64.4 and 23.0%, respectively.

**FIGURE 6:**
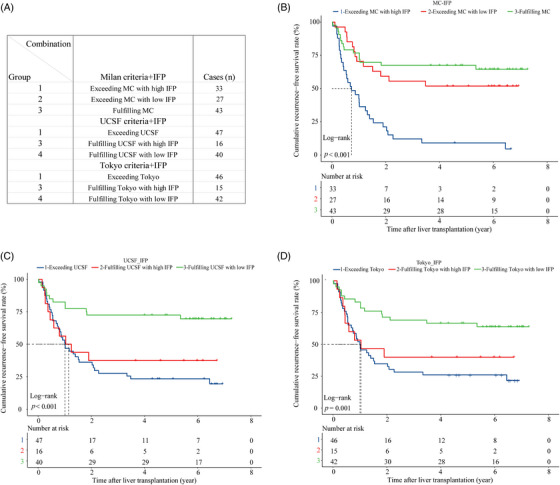
The combination of the IFP and traditional selection criteria predicts posttransplant HCC recurrence in the validation cohort. (A) The combination of the IFP and the traditional candidate selection criteria and subgrouping arrangement. (B) RFS curve for the subgroups of the IFP and Milan criteria combination in the validation cohort. (C) RFS curve for the subgroups of the IFP and the UCSF criteria combination in the validation cohort. (D) RFS curve for the IFP and Tokyo criteria combination subgroups in the validation cohort.

A total of 103 patients were included in the validation cohort. The AUROC of the IFP in predicting posttransplant HCC recurrence was 0.807 (Figure 5A), and the time‐dependent ROC curve showed that the predictive capacity of the IFP increased over time (Figure 5B). The 1, 2, and 3‐year AUROC was shown in Figure S3F separately. The overall C‐index was 0.681, indicating satisfactory predictive discrimination of the IFP. Figure 5C showed the calibration curve of the IFP in predicting 3‐year RFS in the validation cohort, and the Brier score was 0.198 (95% CI, 0.169–0.227) (Figure S4B), indicating good predictive accuracy of the IFP. Recipients in the validation cohort were divided into the low IFP group (*n* = 58) and high IFP group (*n* = 45); survival analysis indicated that recipients in the high IFP group had worse RFS than recipients in the low IFP group (HR:3.61, 95% CI, 2.10–6.30, *p* < 0.001; Figure 5D); the 3‐year recurrence rate was 23.0 and 64.4%, respectively. The combination of the IFP and the aforementioned traditional candidate selection criteria performed well in the recurrence risk stratification of recipients in the validation cohort. Due to the limited sample size of the validation cohort, we divided the population into three subgroups based on the criteria and the IFP as follows (Figure 6A): (1) “MC+IFP” ① Group 1: exceeding MC with high IFP (*n* = 33); ② Group 2: exceeding MC with low IFP (*n* = 27); ③ Group 3: fulfilling MC (*n* = 43). (2) “UCSF+IFP” ① Group 1: exceeding UCSF (*n* = 47); ② Group 2: fulfilling UCSF with high IFP (*n* = 16);③ Group 3: fulfilling UCSF with low IFP (*n* = 40). (3) “Tokyo+IFP” ① Group 1: exceeding Tokyo (*n* = 46); ② Group 2: fulfilling Tokyo with high IFP (*n* = 15); ③ Group 3: fulfilling Tokyo with low IFP (*n* = 42). Nearly half of recipients who exceeded the Milan criteria had low IFP and better RFS, close to that of patients who fulfilled the criteria with low IFP (Figure 6B); the 3‐year RFS rates were 55.6 and 67.4%. Recipients fulfilled the UCSF criteria, and the Tokyo criteria with high IFP had poorer RFS, close to that of recipients who exceeded the criteria (Figure 6C,D). The 3‐year RFS rate was 37.5 versus 27.7% and 40.0 versus 28.3%, respectively. These results indicated that the IFP could also refine the recurrence risk stratification for recipients in the validation cohort.

In accordance with our previous findings in the training cohort, the IFP also performed well in predicting the posttransplant OS of recipients in the validation cohort with AUROC at 0.750 (Figure S6C). Recipients with low IFP (*n* = 58) had better OS than recipients with high IFP (*n* = 45, HR: 3.06, 95% CI: 1.74–5.40, *p* < 0.001; Figure S6D); the 3‐year OS rate was 72.4 versus 40.0%. In addition, we applied propensity score matching (PSM) to the low IFP (*n* = 182) and high IFP (*n* = 111) groups in the whole studied population (*n* = 293). Before matching, the tumor characteristics, including number, single and total diameter, pretransplant AFP levels, and liver functions, were significantly different in the two groups (Table S2). There were 81 recipients in each group after matching, and the characteristics with significant differences were balanced (Table S2). In the 162 matched recipients, the AUROC of the IFP in predicting posttransplant HCC recurrence was 0.812 (Figure S7A), and the C‐index was 0.734, indicating satisfactory predictive discrimination of the IFP. Besides, the calibration curve of the IFP in predicting 3‐year RFS was shown in Figure S7B, and the Brier score was 0.191 (95% CI, 0.163–0.218; Figure S7C), indicating good predictive accuracy. Recipients in the validation cohort were divided into the high IFP group (*n* = 81) and low IFP group (*n* = 81); survival analysis indicated that recipients in the high IFP group had worse RFS than recipients in the low IFP group (HR:5.19, 95% CI, 3.25–8.28, *p* < 0.001; Figure S7D); the 3‐year recurrence rate was 31.9 and 80.1%, respectively.

## 3 DISCUSSION

Posttransplant tumor recurrence is the greatest enemy threatening the long‐term outcomes of patients who receive LT to eliminate HCC radically.^25^, ^26^ It should be emphasized that the OS after HCC recurrence is approximately 1 year,^27^ highlighting the importance of pretransplant risk assessment and timely intervention. The management of LT for patients with HCC is a continuous process, and most recipients need lifelong care by professionals. Inspired by a series of studies focusing on posttransplant recurrence prediction models,^28^, ^29^ we believe an accurate assessment of the recipient's inflammatory status is vital in recurrence prediction. The main underlying reason might be that the complex immune interaction between tumor and host plays an essential role in posttransplant HCC recurrence.^30^, ^31^ In this study, we identified independent risk factors for posttransplant HCC recurrence from pretransplant inflammatory and tumoral biomarkers of 190 recipients. We established an IFP based on three inflammatory biomarkers (IL6, osteocalcin, SII) and a tumor marker AFP. The AUROC and C‐index of the IFP in predicting posttransplant recurrence were 0.792, 0.736 and 0.807, 0.681 in the training and validation cohort, respectively, indicating good predictive discrimination. The Brier score of the IFP in predicting 3‐year RFS was 0.178 and 0.198 in the training and validation cohorts, respectively, showing satisfactory predictive accuracy. Recipients in the low IFP group (IFP ≤ 1.7) had significantly better RFS than those in the high IFP group (IFP > 1.7).

From the peripheral inflammatory spectrum, this study mainly focuses on several cytokine families (TNF superfamily, TGF‐β family, IFN family, Treg cytokines, and MMPs) that are closely related to tumor immunity and play essential roles in tumor formation, development, and prognosis of HCC.^32^, ^33^ IL6 is the most relevant cytokine when considering the diagnosis, progression, and prognosis evaluation of HCC.^34^, ^35^ It has been reported that IL6 is one of the core‐signaling molecules involved in the interaction among HCC cells and their microenvironment, which is consistent with the finding in our study that IL6 is at the center of the PPI network (Figure 1C) constructed by recurrence‐related biomarkers.^36^, ^37^ Recent studies show that in the treatment of HCC, the combination of IL6 blockers and immunosuppressants can improve the tumor microenvironment, enhance the antitumor effect, and reduce the side effects caused by immunosuppressants.^36^ According to the multivariate COX regression analysis, osteocalcin, as a member of the TGF‐β family,^38^ is the only “protective” independent risk factor in the IFP. In addition to maintaining glucose homeostasis, fertility regulation, and cognitive function regulation, osteocalcin also functions as an anti‐inflammatory molecule and biomarker of several solid tumors.^39^, ^40^ Li et al.^41^ reported that osteocalcin can inhibit the secretion of TNF‐α, IL6, and other cytokines by macrophages and reduce the inflammatory response of LPS‐stimulated mice. Genetic variation of osteocalcin gene rs1800247 polymorphism has been found to be a risk factor for HBV‐related HCC.^42^ This study is the first to demonstrate that osteocalcin is a sensitive biomarker of HCC recurrence after LT, and recipients with high pretransplant osteocalcin levels have better RFS.

In the clinical context, SII and AFP are inflammatory and tumoral biomarkers commonly used. The SII proposed by Chinese researchers has been recognized as a convenient and effective index of systemic inflammatory status, which accurately predicts tumor recurrence after hepatectomy for patients with HCC.^43^, ^44^, ^45^ In China, most of the HCC cases have been accompanied by HBV infection for a long time before surgery, and these patients develop special inflammatory environments.^46^ Therefore, including SII in the IFP can comprehensively reflect the inflammatory status of recipients and provide helpful information. After decades of clinical practice, AFP is still the most commonly used serological marker of HCC recurrence after treatment.^47^, ^48^ Including AFP makes the IFP more reliable and accessible to promote recurrence risk assessment. It can be concluded that all four IFP markers help monitor the human body's inflammatory‐immunological balance and have reliable prognostic potential for posttransplant HCC recurrence.

To provide better elucidation for the underlying predictive mechanisms of the IFP, we investigate its relationship with immune cell functional status because recipients have developed unique inflammatory‐immunological homeostasis. We initially identified two distinct pretransplant immune classifications of recipients: “resting” and “activated.” These are the pioneering work in the immune classifications proposed specifically for HCC‐related LT recipients despite plenty of immune classifications for patients with HCC have existed.^49^ We find that recipients in the activated classification have significantly higher levels of 10 T cell subsets, which are influential in antitumor immunity. They also have better RFS than that of recipients in the resting classification, indicating that impaired pretransplant T cell function might be an essential cause of posttransplant HCC recurrence and be reflected by inflammatory status, which the IFP could evaluate. The above findings provide a supportive and scientific explanation for the predictive capacity of the IFP and make it more persuasive to adopt the IFP as a reliable risk stratification tool.

Furthermore, we explore the clinical application value of the IFP by comparing the IFP's accuracy and superiority with the three traditional candidate selection criteria, including the Milan, UCSF, and Tokyo criteria. The results show that IFP had significant advantages in predicting posttransplant HCC recurrence for recipients included in this study. More importantly, when combined with the above criteria, the IFP could screen out the potential beneficiaries of LT from the patients with HCC who have exceeded the criteria (about 50%). It can also identify the recipients with high recurrence risk even if they meet the criteria (about 25%). Thus, the IFP supplements the traditional criteria by incorporating the recipients’ inflammatory and underlying immunological characteristics into the recurrence risk assessment. It is also important to transform the IFP into clinical practice by assisting comprehensive and accurate recurrence risk stratification. We proposed a schematic diagram to provide instruction for the IFP's application in the clinical context (Figure S8).

Although the IFP has good predictive capacity in our research and has significant clinical application value, this study still has shortcomings. First, this is a retrospective study with a relatively limited sample size, and there might be selection bias. We observed that the liver function of recipients at baseline and the recurrence rate varies between the training and validation cohorts. The MELD score and GGT discrepancy might derived from demographic changes of patient resources in the two centers. In addition, patients in the training cohort were from a more recent time and had received more comprehensive medical care, which contributed to better liver function and lower MELD scores. Prolonged follow‐up time might be associated with increased recurrence cases. We performed PSM in the overall studied population to alleviate selection bias. The predictive capacity of the IFP was verified to be consistent after PSM. To reduce the influence of confounding factors, we carried out multivariate COX regression analysis, which was promoted as a method for controlling confounding bias. Being constrained by sample availability, we carried out temporal validation as external validation, which still needs to be optimized by launching multicenter prospective studies in different medical centers. It also should be noted that we carried out FC detection of pretransplant samples on 101 instead of 190 recipients in the training cohort because (1) the detection was performed based on the professional advice from transplant clinicians and the individual desires of recipients; (2) some of the samples failed the quality check and relative results were not eligible for further analysis. Despite the predictive discrimination and calibration of the IFP being good in the current study, high‐quality, large‐scale validation is still needed for verifying and optimizing the IFP in further clinical applications.

In summary, the recurrence prediction model IFP, based on the pretransplant peripheral IL6, osteocalcin, SII, and AFP levels, performed well in predicting posttransplant HCC recurrence. When combined with traditional candidate selection criteria, the IFP could optimize the recurrence risk stratification for HCC‐related LT.

## 4 MATERIALS AND METHODS

### 4.1 Study population

A total of 293 patients underwent LT from donation after cardiac death in two independent medical centers, the First Affiliated Hospital of Zhejiang University School of Medicine and Shulan (Hangzhou) Hospital, from January 2015 to December 2019 were enrolled in this retrospective study. The operations were performed by surgeons who had received identical surgical training and were certified. The inclusion criteria were as follows: (1) a complete preoperative ultrasound, computed tomography (CT), or magnetic resonance imaging (MRI) scan examination of the liver imaging data, (2) a posttransplant histopathology report of HCC on the explant liver, and (3) recipients who were older than 18 years. The exclusion criteria were (1) other types of liver cancer instead of HCC proven by posttransplant pathology, (2) tumor thrombus in the main truck of portal vein reported by pretransplant imaging, (3) OS less than 30 days, (4) patients who underwent secondary LT or multiorgan transplantation, and (5) patients with incomplete medical records or unavailable blood sample. In the current study, two batches of plasma collected from blood samples were sent for cytokine profiling. In detail, the plasma samples collected from January 1, 2018 to December 31, 2019 were set into batch 1 (the training cohort, *n* = 190) to ensure the data quality. The plasma samples collected from January 1, 2015 to December 31, 2017 were set into batch 2 (the validation cohort, *n* = 103) to testify the predictive capacity of our prediction model in samples stored for a long period.

Figure 1A showed the schematic illustration of our research. The study was designed in compliance with the STROBE and TRIPOD guidelines.^50^, ^51^

### 4.2 Data collection and follow‐up

Clinical data were collected from the CLTR, strictly according to the Regulations on Human Organ Transplant and national legal requirements, including age, gender, body mass index (BMI), MELD score, HBV infection, cirrhotic status, pretransplant downstaging therapy or liver resection (DS/LR), tumor number, the maximum and total diameter of tumor, pretransplant AFP level, blood routine test results, and follow‐up information. Among them, cirrhosis status, tumor number, and the maximum and total diameter of the tumor were derived from pretransplant CT or MRI, and the pathological diagnosis of HCC was confirmed by explant histopathology. The grouping of age, BMI, MELD score, and tumor features was in reference to previous studies.^52^, ^53^ According to previously reported literature, similar pretransplant and posttransplant management strategies were utilized in the two centers.^52^ Patients were divided into different groups by three traditional candidate selection criteria: Milan criteria, UCSF criteria, and Tokyo criteria, as reported previously. Patients were regularly followed up in outpatient clinics, and serum AFP, ultrasound, and CT (every 3 months for the first year, every 6 months for the next year, and then annually) were the main items of the recurrence monitoring. The primary endpoint for this study was death due to any cause or the last follow‐up (loss to follow‐up).

### 4.3 Cytokine and T‐cell profiling of pretransplant blood sample

#### 4.3.1 Cytokine profiling

1. Plasma was obtained at the admission time before transplantation by centrifuging the blood samples for 1 min at 1000 *g* and diluted fourfold in Bio‐Plex Sample Diluent HB buffer for cytokine profiling.
2. Per the manufacturer's instructions, the diluted plasma samples were analyzed via a Bio‐Plex Pro Human Cytokine Panel 37‐Plex assay (Bio‐Rad; Cat# 171AL001M).
3. The samples were analyzed using the Bio‐Plex system and Bio‐Plex Manager software with 5PL curve fitting (Bio‐Rad Laboratories). The reader was set to read a minimum of 200 beads, and the results were expressed as median fluorescence intensity.

#### 4.3.2 T‐cell profiling

The expression levels of 32 T‐cell subsets in pretransplant peripheral blood samples of recipients were monitored by FC.

1. The blood sample collection and FC detection process were in strict accordance with the Guidelines for Peripheral Lymphocyte Subsets by FC.
2. Each sample was divided into tube 1 for detecting total T cell, cytotoxic T cell (Tc) subsets, and helper T cell (Th) subsets, and into tube 2 for detecting regulatory T cell (Treg) subsets, γδT cell, Th1 cell, Th2 cell, and Th17 cell subsets.
3. Different T cell subsets were identified by their surficial molecules, as shown in Table S1.
4. Because the same sample was detected in two tubes, the results of tube 2 were adjusted by that of tube 1 as follows: the proportion of each cell subset (with total lymphocytes as the denominator) and the absolute number of each cell subset in tube 2 were obtained by the test value of tube 2 × coefficient *k*, *k* = the total number of lymphocytes in tube 2/the total number of lymphocytes in tube 1.

### 4.4 Statistics

For baseline data analysis and presentation, the Kolmogorov–Smirnov test (K–S test) was applied to test the normality of continuous variables, and the variable did not fit a normal distribution if the *p* value < 0.050. Normally distributed variables were presented as a mean ± standard deviation, and non‐normally distributed variables were presented as the medians (interquartile range), and categorical variables were expressed as numbers (percentages). To balance important patient characteristics between groups and reduce selection bias, we implemented stratified and PSM, and the caliper value was set to 0.2. RFS was defined as the time from LT to the tumor return, death led by all causes, or the last follow‐up conducted. For differential analysis between the training and validation cohorts, Student's *t*‐test was utilized to analyze normally distributed continuous variables, and the Mann–Whitney test or Wilcoxon rank sum test was used for non‐normally distributed continuous variables; Pearson's chi‐square test or Fisher's exact test was applied to analyze categorical variables. For subsequent analyses, all concentration data were natural log‐transformed and standardized. We carried out univariate and multivariate Cox proportional hazards regression analysis to identify independent prognostic factors in a stepwise mode. The risk model for predicting tumor recurrence after LT was developed based on the coefficients of independent prognostic factors derived from the multivariate Cox proportional hazards regression analysis. The survival curve was analyzed using the KM method, and comparisons between groups were performed using the log‐rank test. The ROC curve and concordance index (C‐index) were used to evaluate the predictive discrimination of the model. The calibration curve and Brier score were used to present and evaluate the model's predictive accuracy. To ensure the rigor of the method, the PH assumption of the variables was checked before the performance of the multivariate Cox regression analysis. The NRI and the IDI were calculated to compare the model's predictive capacity with traditional candidate selection criteria, including Milan criteria, UCSF criteria, and Tokyo criteria.^8^, ^54^ Competing risk analysis was performed using the Fine‐Gray model to calculate the hazard of recurrence while accounting for the competing outcome of nonrecurrence mortality. The consensus clustering analysis was used to identify function subtypes of T cells.

Statistical analysis was performed using IBM SPSS Statistics 25.0 (SPSS Inc., Chicago, IL), R‐project (version 3.6.1, https://www.r‐project.org/), GraphPad Prism Software (GraphPad Prism version 9.1 for Windows; GraphPad Software, www.graphpad.com), and Sangerbox tools, a free online platform for data analysis (http://vip.sangerbox.com/). Cluster analysis was performed using the ConsensusClusterPlus package of R, and the optimal number of clusters was determined using the empirical cumulative distribution function plot. The schematic diagram was created with BioRender, a scientific graphic software (www.biorender.com). . A two‐sided *p* value < 0.050 was considered statistically significant.

## AUTHOR CONTRIBUTIONS

*Conception and design*: Xiao Xu, Shusen Zheng, Di Lu, Modan Yang, Zuyuan Lin, Li Zhuang, and Linhui Pan. *Experiment performance and data acquisition*: Modan Yang, Zuyuan Lin, Linhui Pan, Li Zhuang, Siyi Dong, Junli Chen, Hao Chen, Wei Shen, Chiyu He, and Zhe Yang. *Analysis and interpretation of data*: Modan Yang, Zuyuan Lin, Linhui Pan, Li Zhuang, Di Lu, Rui Wang, Renyi Su, Qinfen Xie, and Junjie Yin. *Writing, review, and/or revision of the manuscript*: Modan Yang, Zuyuan Lin, Di Lu, Jianyong Zhuo, Xinyu Yang, Xuyong Wei, Zhihang Hu, Huigang Li, and Junjie Yin. All authors read and approved the final manuscript.

## CONFLICT OF INTEREST STATEMENT

The authors declare that they have no known conflict of financial interests or personal relationships that could have appeared to influence the work reported in this paper.

## ETHICS STATEMENT

The Institutional Ethical and Scientific Review Board of the First Affiliated Hospital, School of Medicine, Zhejiang University, and the Shulan (Hangzhou) Hospital approved the study protocol (approval number: Scientific Research Quick Review No. 147, 2017). Clinical Study Approval Form of the Research Ethics Committee: KY2021014). All patients were informed ahead of transplantation and their consents were recorded. The whole project was performed under the guidelines of the 1975 Declaration of Helsinki, the Ethics Committee of the corresponding hospitals, and the Organ Transplant Committee of China. We claim that none of the organs came from executed prisoners. All authors had access to the study data and reviewed and approved the final manuscript.

## Supporting information

Supporting Information

## Data Availability

The data generated in this study are not publicly available due to information that could compromise patient privacy or consent but are available upon reasonable request from the corresponding author.
