# Supplementary material for: An inflammatory liquid fingerprint predicting tumor recurrence after liver transplantation for hepatocellular carcinoma
Source: MedComm (2020). 2024 Aug 26;5(9):e678. doi: 10.1002/mco2.678 (PMC11345533; doi:10.1002/mco2.678)
Supplement: Supplementary file 1 — Supporting Information [file MCO2-5-e678-s001.docx]

**Title Page**

**Title**

An inflammatory liquid fingerprint predicting tumor recurrence after liver transplantation for hepatocellular carcinoma

**Running head**

A model predicts posttransplant HCC recurrence

**Authors and affiliations**

Modan Yang^1,2#^, Zuyuan Lin^3,4#^, Li Zhuang^5#^, Linhui Pan^6^, Rui Wang^4^, Hao Chen^4^, Zhihang Hu^4^, Wei Shen^4^, Jianyong Zhuo^6^, Xinyu Yang^3,4^, Huigang Li^4^, Chiyu He^4^, Zhe Yang^5^, Qinfen Xie^5^, Siyi Dong^7^, Junli Chen^7^, Renyi Su^4^, Xuyong Wei^8,2^, Junjie Yin^6^, Shusen Zheng^7,2,4,5*^, Di Lu^8,2*^, Xiao Xu^9,10,2*^

^#^ The first three authors contributed equally to this article.

^*^ Professor Xiao Xu is the corresponding author. Professor Shusen Zheng and Dr. Di Lu are co-corresponding authors.

^1^ Department of Breast Surgery, The Second Affiliated Hospital, Zhejiang University School of Medicine, Hangzhou, 310009, China

^2^ NHC Key Laboratory of Combined Multi-organ Transplantation, Hangzhou, 310003, China

^3^ Key Laboratory of Integrated Oncology and Intelligent Medicine of Zhejiang Province, Hangzhou First People’s Hospital, Hangzhou, 310006, China

^4^ Zhejiang University School of Medicine, Hangzhou 310058, China

^5^ Department of Hepatobiliary and Pancreatic Surgery, Shulan (Hangzhou) Hospital, Hangzhou, 310022, China

^6^ Department of Hepatobiliary and Pancreatic Surgery, Affiliated Hangzhou First People's Hospital, School of Medicine, Westlake University, Hangzhou, 310006, China

^7^ National Center for Healthcare Quality Management in Liver Transplant, Hangzhou, 310003, China

^8^ Department of Hepatobiliary & Pancreatic Surgery and Minimally Invasive Surgery, Zhejiang Provincial People's Hospital, Affiliated People's Hospital, Hangzhou Medical College, Hangzhou, 310014, China

^9^ School of Clinical Medicine, Hangzhou Medical College, Hangzhou, 310059, China

^10^ Institute of Translational Medicine, Zhejiang University School of Medicine, Hangzhou, 310000, China

**Corresponding Author:**

Xiao Xu, School of Clinical Medicine, Hangzhou Medical College, Hangzhou, China. Email: zjxu@zju.edu.cn.

Di Lu, Department of Hepatobiliary & Pancreatic Surgery and Minimally Invasive Surgery, Zhejiang Provincial People's Hospital, Affiliated People's Hospital, Hangzhou Medical College, Hangzhou, Zhejiang, China. Email: zjuludi@zju.edu.cn.

Shusen Zheng, Department of Hepatobiliary and Pancreatic Surgery, the First Affiliated Hospital, Zhejiang University School of Medicine, Hangzhou, China. Email: shusenzheng@zju.edu.cn.

**Supplementary material**

**Contents**

**Supplementary table4**

Table S1. Immune cell phenotype 4

Table S2. Baseline characteristics of low-IFP and high-IFP patients 5

**Supplementary figure6**

Figure S1. The test of proportional hazards assumption for the forty-four inflammatory and tumoral biomarkers6

Figure S2. ROC and KM analysis of the four independent risk factors in predicting posttransplant HCC recurrence in the training cohort 7

Figure S3. Comparison of the four independent biomarkers and 1,2,3-year ROC curve for the IFP in predicting posttransplant HCC recurrence in the training and validation cohort...8

Figure S4. The Brier score of the IFP and competing-risk model for the IFP to evaluate the competing risk of non-recurrence death9

Figure S5. The RFS curves and IDI plots for the traditional criteria in the training cohort10

Figure S6. The IFP predicts OS after liver transplantation for HCC in the training and validation cohorts.11

Figure S7. The IFP predicts posttransplant HCC recurrence in the whole population after propensity score matching.12

Figure S8. The schematic diagram of the IFP in the clinical context.13

**Supplementary table**

**Table S1. Immune cell phenotype**

| Immune cell | Phenotype | Number  (count/ul)^†^ |
| --- | --- | --- |
| Total T cell | CD3^+^ | 386 (241-539) |
| CD4^+^ T cell | CD3^+^ 、CD4^+^ 、CD8^-^ | 230 (137-324) |
| CD8^+^ T cell | CD3^+^ 、CD8^+^ 、CD4^-^ | 118 (68-189) |
| Double positive T cell（DPT, CD4^+^ CD8^+^ ） | CD3^+^ 、CD4^+^ 、CD8^+^ | 2 (1-5) |
| Double negative T cell（DNT, CD4^+^ CD8^+^ ） | CD3^+^ 、CD4^-^ 、CD8^-^ | 27 (16-44) |
| Primary CD4^+^ T cell | CD3^+^ 、CD4^+^ 、CD8^-^ 、CD45RA^+^ 、CCR7^+^ | 54 (30-88) |
| Central memory CD4^+^ T cell | CD3^+^ 、CD4^+^ 、CD8^-^ 、CD45RA^-^ 、CCR7^+^ | 72 (39-107) |
| Effector CD4^+^ T cell | CD3^+^ 、CD4^+^ 、CD8^-^ 、CD45RA^+^ 、CCR7^-^ | 3 (1-6) |
| Effector memory CD4^+^ T cell | CD3^+^ 、CD4^+^ 、CD8^-^ 、CD45RA^-^ 、CCR7^-^ | 74 (48-130) |
| Activated CD4^+^ T cell | CD3^+^ 、CD4^+^ 、CD8^-^ 、CD38^+^ 、HLA- DR^+^ | 8 (4-13) |
| Primary CD8^+^ T cell | CD3^+^ 、CD4^-^ 、CD8^+^ 、CD45RA^+^ 、CCR7^+^ | 22 (11-45) |
| Central memory CD8^+^ T cell | CD3^+^ 、CD4^-^ 、CD8^+^ 、CD45RA^-^ 、CCR7^+^ | 2 (1-5) |
| Effector CD8^+^ T cell | CD3^+^ 、CD4^-^ 、CD8^+^ 、CD45RA^+^ 、CCR7^-^ | 34 (17-73) |
| Effector memory CD8^+^ T cell | CD3^+^ 、CD4^-^ 、CD8^+^ 、CD45RA^-^ 、CCR7^-^ | 34 (20-65) |
| Activated CD8^+^ T cell | CD3^+^ 、CD4^-^ 、CD8^+^ 、CD38^+^ 、HLA- DR^+^ | 7 (4-16) |
| Primary DPT cell | CD3^+^ 、CD4^+^ 、CD8^+^ 、CD45RA^+^ 、CCR7^+^ | 0 (0-1) |
| Central memory DPT cell | CD3^+^ 、CD4^+^ 、CD8^+^ 、CD45RA^-^ 、CCR7^+^ | 0 (0-1) |
| Effector DPT cell | CD3^+^ 、CD4^+^ 、CD8^+^ 、CD45RA^+^ 、CCR7^-^ | 0 (0-1) |
| Effector memory DPT cell | CD3^+^ 、CD4^+^ 、CD8^+^ 、CD45RA^-^ 、CCR7^-^ | 1 (0-3) |
| Activated DPT cell | CD3^+^ 、CD4^+^ 、CD8^+^ 、CD38^+^ 、HLA-DR^+^ | 2 (0-1) |
| Primary DNT cell | CD3^+^ 、CD4^-^ 、CD8^-^ 、CD45RA^+^ 、CCR7^+^ | 2 (1-3) |
| Central memory DNT cell | CD3^+^ 、CD4^-^ 、CD8^-^ 、CD45RA^-^ 、CCR7^+^ | 0 (0-1) |
| Effector DNT cell | CD3^+^ 、CD4^-^ 、CD8^-^ 、CD45RA^+^ 、CCR7^-^ | 12 (6.24) |
| Effector memory DNT cell | CD3^+^ 、CD4^-^ 、CD8^-^ 、CD45RA^-^ 、CCR7^-^ | 8 (4-16) |
| Activated DNT cell | CD3^+^ 、CD4^-^ 、CD8^-^ 、CD38^+^ 、HLA-DR^+^ | 2 (1-4) |
| Th1 cell | CD3^+^ 、CD4^+^ 、CD183^+^ 、CD196^-^ | 45 (24-72) |
| Th2 cell | CD3^+^ 、CD4^+^ 、CD183^-^ 、CD196^-^ | 89 (55-133) |
| Th17 cell | CD3^+^ 、CD4^+^ 、CD183^-^ 、CD196^+^ | 29 (19-47) |
| Total Treg cell | CD3^+^ 、CD4^+^ 、CD25^+^ 、CD127^low^/^-^ | 18 (11-26) |
| Memory Treg cell | CD3+ 、CD4+ 、CD25+ 、CD127^low^/^-^ 、CD194+ | 12 (7-17) |
| Primary Treg cell | CD3^+^ 、CD4^+^ 、CD25^+^ 、CD127^low^/^-^ 、CD194^-^ | 6 (3-8) |
| Activated Treg cell | CD3^+^ 、CD4^+^ 、CD25^+^ 、CD127^low^/^-^ 、HLA-DR^+^ | 5 (3-7) |
| γδT cell | CD3^+^ 、TCRγδ^+^ | 11 (4-19) |

**Footnotes †** Non-normally distributed continuous variables were presented as medians (IQR, interquartile range).

| **Table S2. Baseline characteristics of low-IFP and high-IFP patients** | | | | | | |
| --- | --- | --- | --- | --- | --- | --- |
| **Characteristics** | **Before propensity score matching** | | | **After propensity score matching** | | |
|  | **Low-IFP**  **n(%)**^†^ | **High-IFP**  **n(%)** | ***p* value^*^** | **Low-IFP**  **n(%)** | **High-IFP**  **n(%)** | ***p* value** |
| **Total** | 182(62.1%) | 111(27.9%) |  | 81(50.0%) | 81(50.0%) |  |
| **Age(year)** |  |  | 0.488 |  |  | 0.868 |
| **≤ 50** | 60(33.0%) | 41(36.9%) |  | 28(34.6%) | 27(33.3%) |  |
| **＞50** | 122(67.0%) | 70(63.1) |  | 53(65.4%) | 54(66.7%) |  |
| **Gender** |  |  | 0.893 |  |  | 0.786 |
| **Male** | 166(91.2%) | 102(91.9%) |  | 73(90.1%) | 74(91.4%) |  |
| **Female** | 16(8.8%) | 9(8.1%) |  | 8(9.9%) | 7(8.6%) |  |
| **BMI** |  |  | 0.072 |  |  | 0.374 |
| **≤ 25** | 125(68.7%) | 87(78.4%) |  | 57(70.4%) | 62(76.5%) |  |
| **＞25** | 57(31.3%) | 24(21.6%) |  | 24(29.6%) | 19(23.5%) |  |
| **Hepatitis B virus infection** |  |  | 0.279 |  |  | 1.000 |
| **No** | 24(13.2%) | 10(9.0%) |  | 9(11.1%) | 9(11.1%) |  |
| **Yes** | 158(86.8%) | 101(91.0%) |  | 72(88.9%) | 72(88.9%) |  |
| **Cirrhosis** |  |  | 0.474 |  |  | 0.650 |
| **No** | 4(2.2%) | 4(3.6%) |  | 3(3.7%) | 2(2.5%) |  |
| **Yes** | 178(97.8%) | 107(96.4%) |  | 78(96.3%) | 79(97.5%) |  |
| **MELD score** |  |  |  |  |  | 0.110 |
| **≤ 26** | 113(62.1%) | 62(55.9%) | 0.291 | 53(65.4%) | 43(53.1%) |  |
| **＞26** | 69(37.9%) | 49(44.1%) |  | 28(34.6%) | 38(46.9%) |  |
| **DS/LR** |  |  | 0.316 |  |  | 0.156 |
| **No** | 76(41.8%) | 53(47.7%) |  | 42(51.9%) | 33(40.7%) |  |
| **Yes** | 106(58.2%) | 58(52.3%) |  | 39(48.1%) | 48(59.3%) |  |
| **Tumor number** |  |  | 0.460 |  |  | 0.431 |
| **Single** | 95(52.2%) | 53(47.7%) |  | 45(55.6%) | 40(49.4%) |  |
| **Multifocal** | 87(47.8%) | 58(52.3%) |  | 36(44.4%) | 41(50.6%) |  |
| **Max tumor diameter(cm)** |  |  | **<0.001** |  |  | 0.398 |
| **≤ 5** | 153(84.1%) | 60(54.1%) |  | 58(71.6%) | 53(65.4%) |  |
| **＞5** | 29(15.9%) | 51(45.9%) |  | 23(28.4%) | 28(34.6%) |  |
| **Total tumor diameter(cm)** |  |  | **<0.001** |  |  | 0.139 |
| **≤10** | 150(82.4%) | 56(50.5%) |  | 57(70.4%) | 48(59.3%) |  |
| **>10** | 32(17.6%) | 55(49.5%) |  | 24(29.6%) | 33(40.7%) |  |
| **AFP(ng/ml)** ^‡^ | 11.6(4.1 - 53.4) | 134.4(7.3 - 849.7) | **<0.001** | 11.0(4.08 - 79.3) | 37.0(5.88 - 335.7) | 0.116 |
| **ALT(U/L)** | 33.3(23.0 - 42.8) | 45.0(29.0 - 62.0) | **0.001** | 31.0(23.0 - 40.9) | 40.3(27.8 - 62.0) | 0.285 |
| **AST(U/L)** | 40.0(27.3 - 54.0) | 51.0(35.0- 99.0) | 0.054 | 40.5(33.3 - 55.5) | 42.3(35.0 - 85.5) | 1.000 |
| **GGT(U/L)** | 60.0(34.0 - 100.8) | 100.0(69.0 - 174.5) | **<0.001** | 68.0(35.5 - 121.3) | 88.0(51.0 - 139.8) | 0.116 |

**Footnotes** † Categorical variables are expressed as the count (n) and proportion (percentage). ‡ Non-normally distributed continuous variables were presented as medians medians (IQR). * Student’s t-test was utilized to analyze normally distributed continuous variables, and the Mann-Whitney test or Wilcoxon rank sum test was used for non-normally distributed continuous variables, Pearson’s chi-square test or Fisher’s exact test was applied to analyze categorical variables. Abbreviations: BMI, body mass index; MELD, end-stage liver disease; DS/LR, downstaging therapy or liver resection; AFP, α-fetoprotein; ALT, alanine aminotransferase; AST, aspartate aminotransferase; GGT, γ-glutamyltranspeptidase

**Supplementary figure**

**
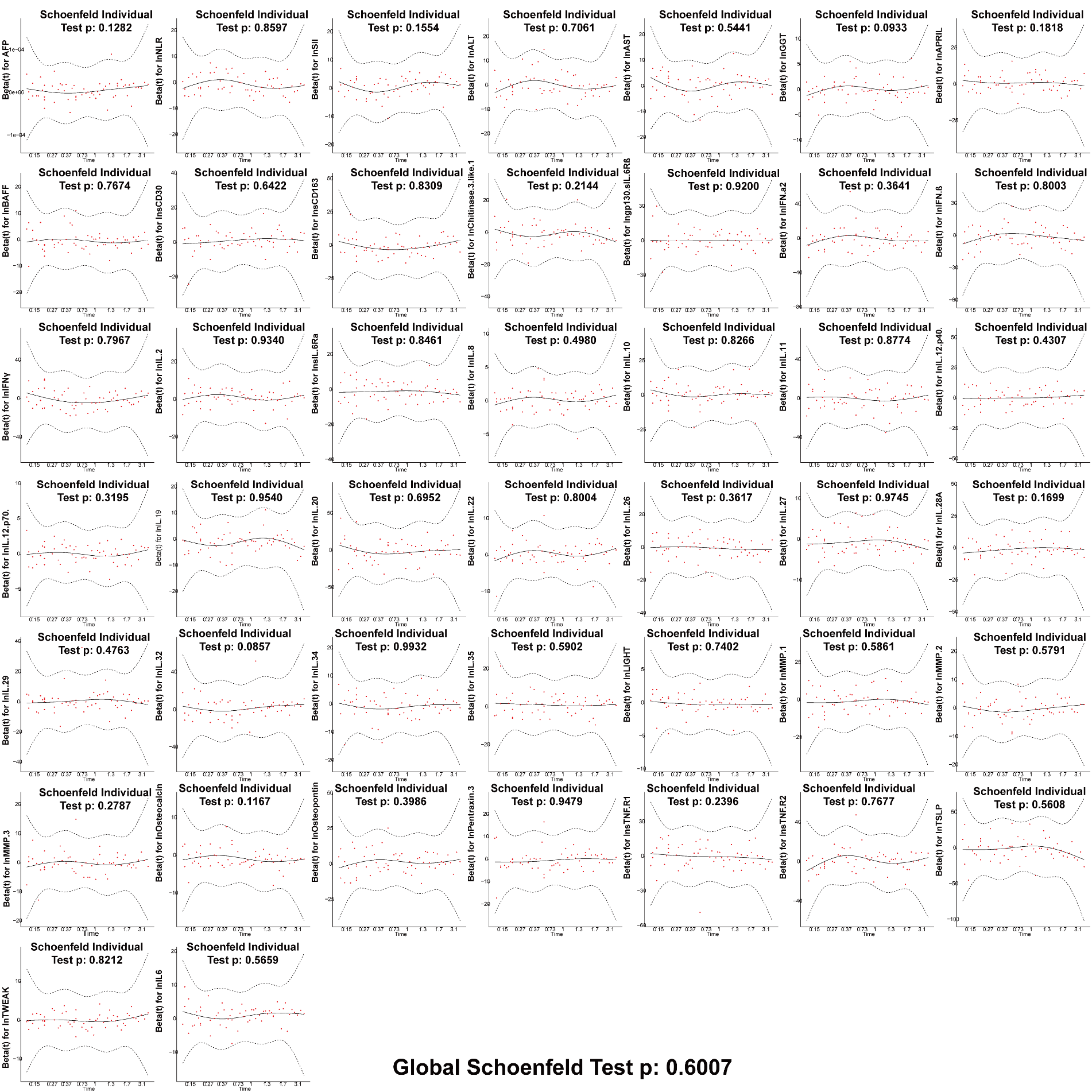
**

**Figure S1.** The test of proportional hazards assumption for the forty-four inflammatory and tumoral biomarkers.

**
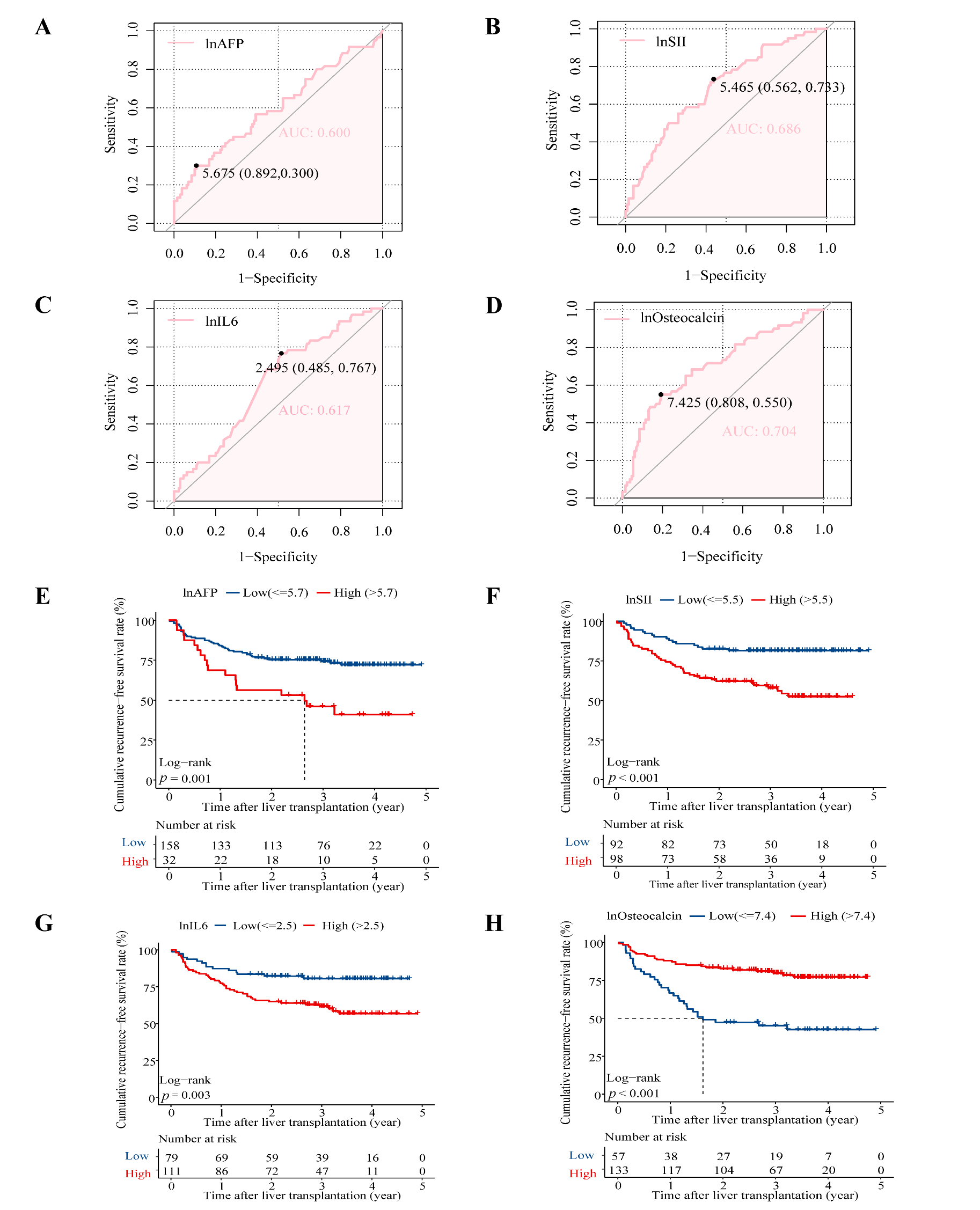
**

**Figure S2. ROC and KM analysis of the four independent risk factors in predicting posttransplant HCC recurrence in the training cohort.** A. ROC curve for lnAFP. The AUROC was 0.600. B. ROC curve for lnSII. The AUROC was 0.686. C. ROC curve for lnIL6. The AUROC was 0.617. D. ROC curve for lnOsteocalcin. The AUROC was 0.704. E. RFS curve for lnAFP. Recipients in the low lnAFP and high lnAFP groups had significantly different RFS (*p =* 0.001). The 3-year RFS rate was 74.3% and 46.0%, respectively. F. RFS curve for lnSII. Recipients in the low lnSII and high lnSII groups had significantly different RFS (*p* < 0.001). The 3-year RFS rate was 81.4% and 57.9%, respectively. G. RFS curve for lnIL6. Recipients in the low lnIL6 and high lnIL6 groups had significantly different RFS (*p* = 0.003). The 3-year RFS rate was 80.5% and 61.6%, respectively. H. RFS curve for lnOsteocalcin. Recipients in the low lnOsteocalcin and high lnOsteocalcin groups had significantly different RFS (*p <* 0.001). The 3-year RFS rate was 45.2% and 79.7%, respectively. RFS: recurrence-free survival. ROC: receiver operating characteristic. AUROC: area under the receiver operating characteristic.

**
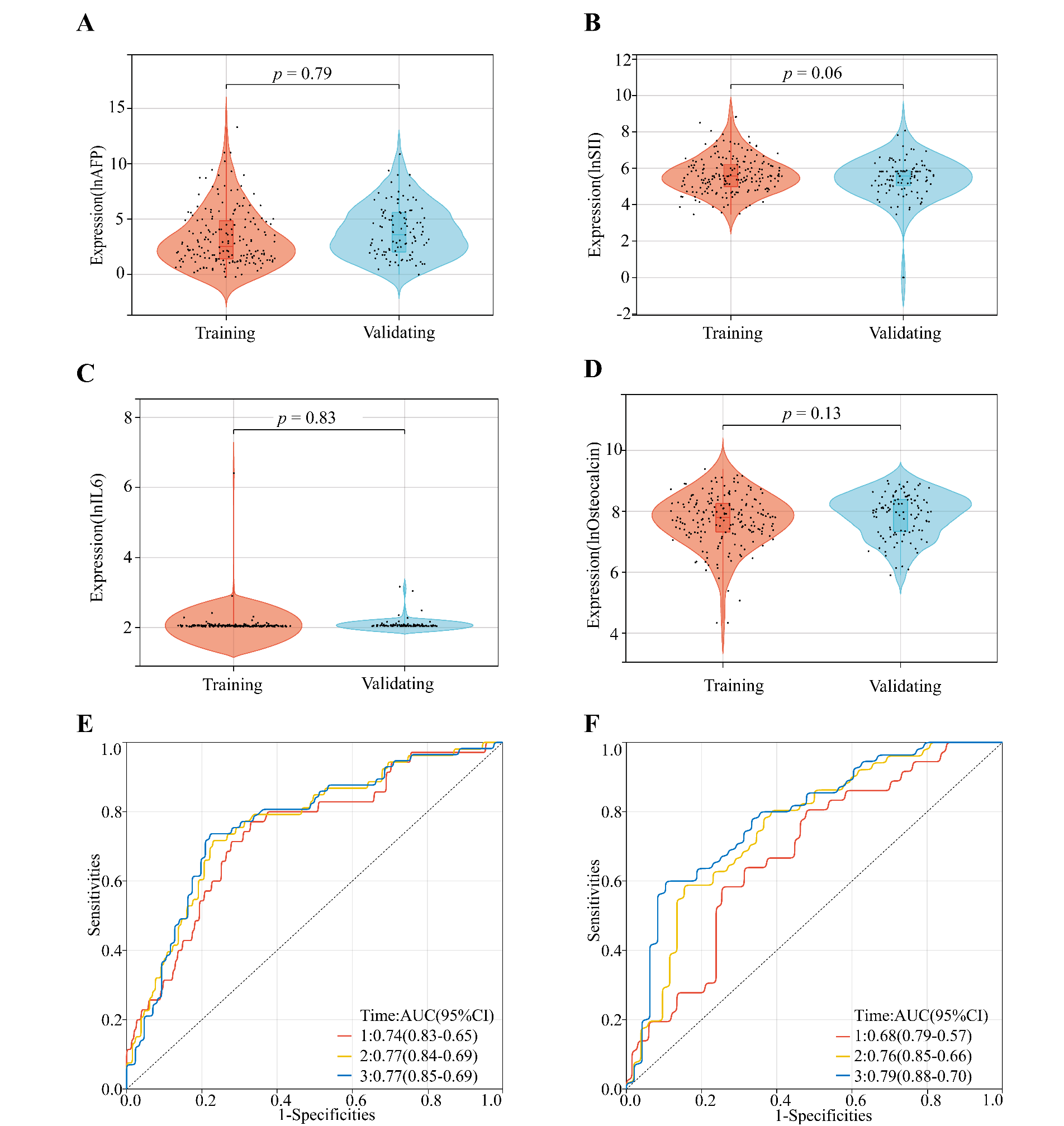
**

**Figure S3. Comparison of the four independent biomarkers in the training and validation cohort.** A. The expression level of AFP (logarithmic) in the training and the validation cohort (*p* = 0.79). B. The expression level of SII (logarithmic) in the training and the validation cohort (*p* = 0.06). C. The expression level of IL6 (logarithmic) in the training and the validation cohort (*p* = 0.83). D. The expression level of osteocalcin (logarithmic) in the training and the validation cohort (*p* = 0.13). E. The 1,2,3-year AUROC of the IFP in predicting posttransplant HCC recurrence in the training cohort was 0.74, 0.77, and 0.77, respectively. F. The 1,2,3-year AUROC of the IFP in predicting posttransplant HCC recurrence in the validation cohort was 0.68, 0.76, and 0.79, respectively.

**
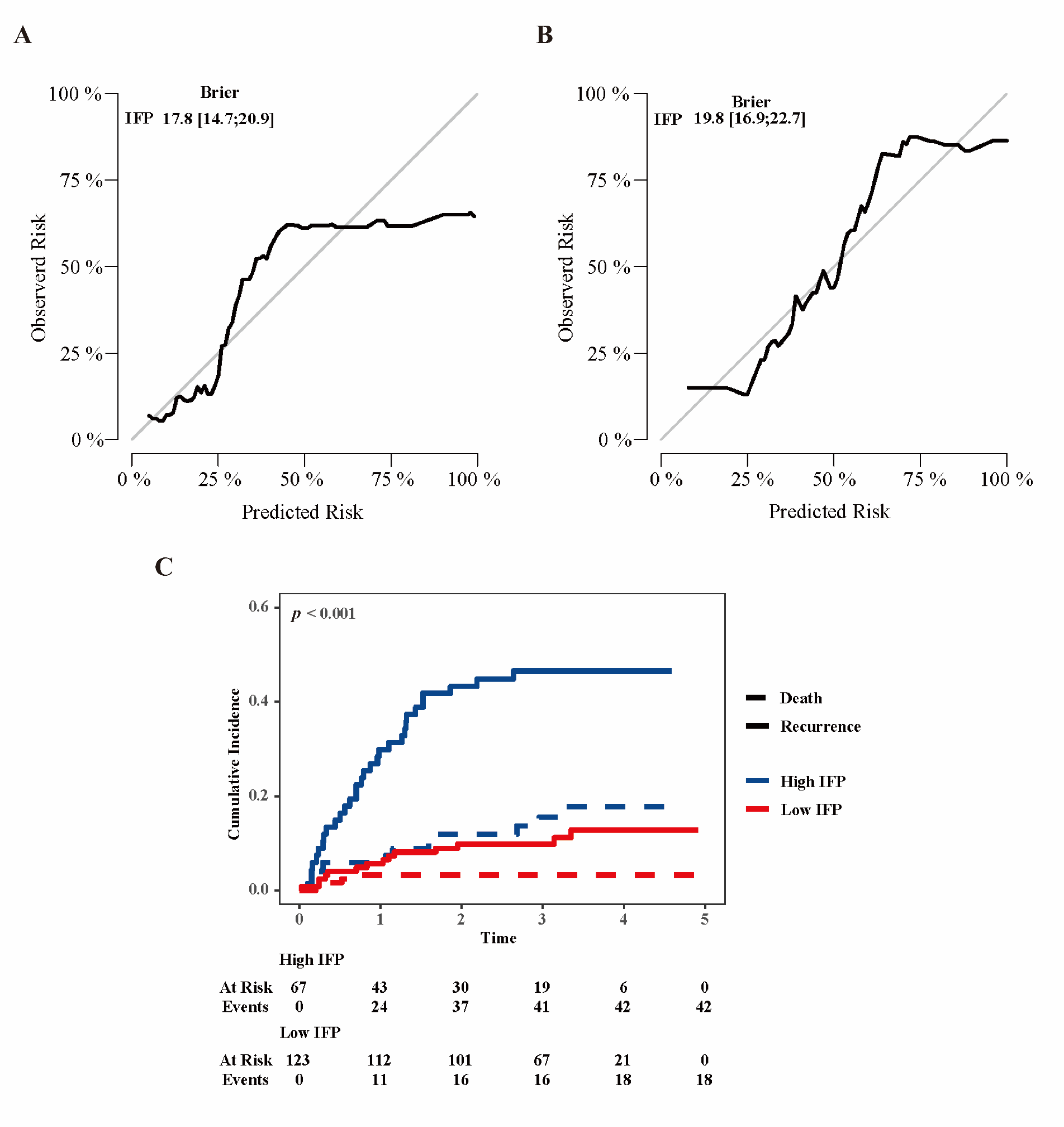
**

**Figure S4.** **The Brier score of the IFP and competing-risk model for the IFP evaluate the competing risk of non-recurrence death.**


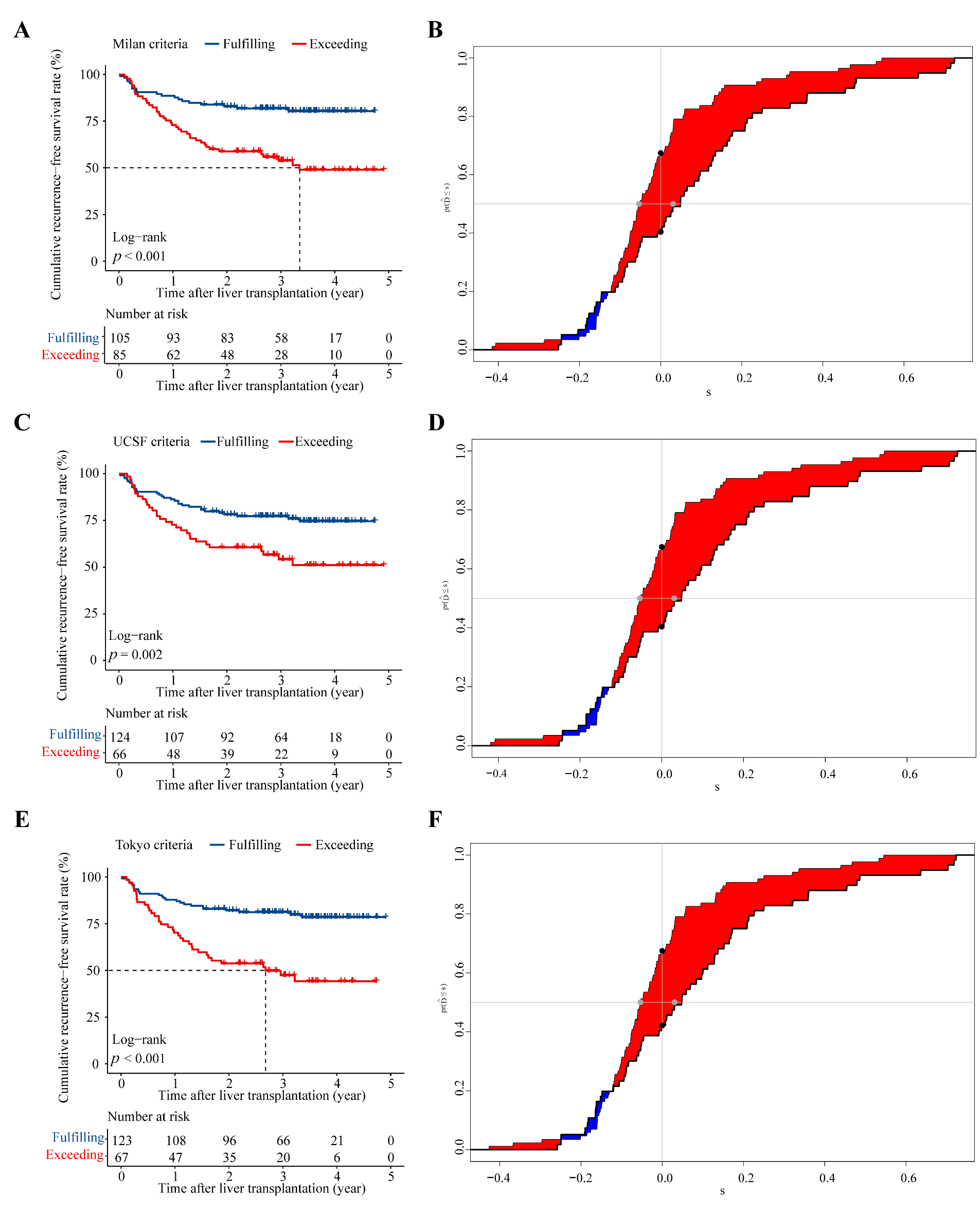


**Figure S5. The RFS curves for the traditional criteria in predicting posttransplant HCC recurrence and IDI plots for the comparison of the IFP and the traditional criteria.** A. RFS curve for the Milan criteria in the training cohort. Recipients who fulfilled and exceeded the criteria had significantly different RFS (*p* < 0.001). The 3-year RFS rate was 81.8% and 53.8%, respectively. B. The IDI plot compares the IFP and the Milan criteria in predicting posttransplant HCC recurrence in the training cohort. The IDI was 10.3% (*p* = 0.040). C. RFS curve for the UCSF criteria in the training cohort. Recipients who fulfilled and exceeded the criteria had significantly different RFS (*p* < 0.001). The 3-year RFS rate was 77.3% and 54.1%, respectively. D. The IDI plot compares the IFP and the UCSF criteria in predicting posttransplant HCC recurrence in the training cohort. The IDI was 10.3% (*p* = 0.044). E. RFS curve for the Tokyo criteria in the training cohort. Recipients who fulfilled and exceeded the criteria had significantly different RFS (*p* < 0.001). The 3-year RFS rate was 81.2% and 47.4%, respectively. F. The IDI plot compares the IFP and the Tokyo criteria in predicting posttransplant HCC recurrence in the training cohort. The IDI was 10.2% (*p* = 0.030). UCSF: University of California, San Francisco. IDI: Integrated Discrimination Improvement.


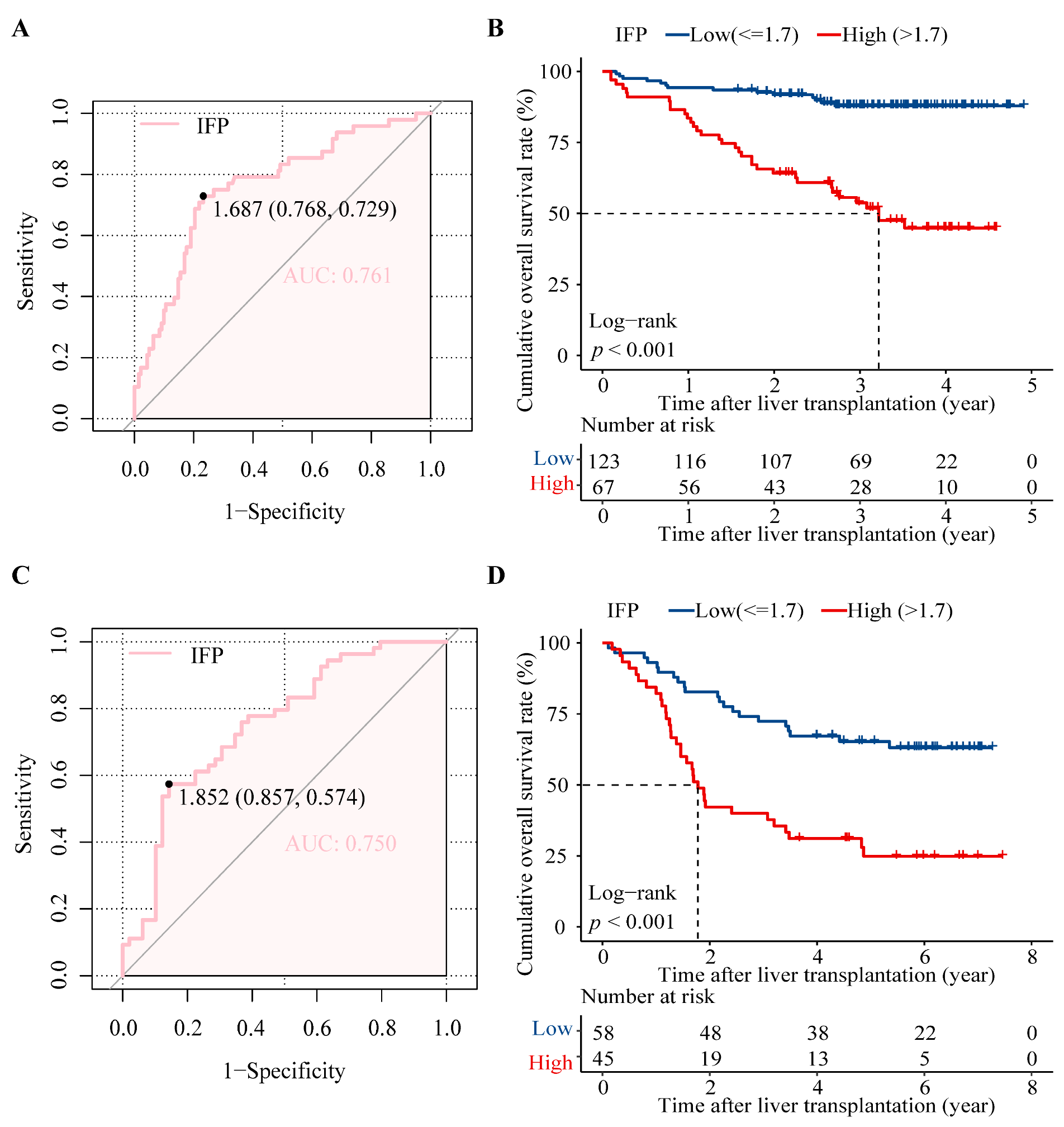


**Figure S6. The IFP predicts OS after liver transplantation for HCC in the training and validation cohorts.** A. ROC curve for the IFP in the training cohort. The AUROC was 0.761. B. OS curve for the IFP in the training cohort. Recipients in the low IFP and high IFP groups had significantly different OS (*p* < 0.001). The 3-year OS rate was 87.9% and 53.8%, respectively. C. ROC curve for the IFP in the training cohort. The AUROC was 0.750. D. OS curve for the IFP in the training cohort. Recipients in the low IFP and high IFP groups had significantly different OS (*p* < 0.001). The 3-year OS rate was 72.4% and 40.0%, respectively. OS: overall survival.


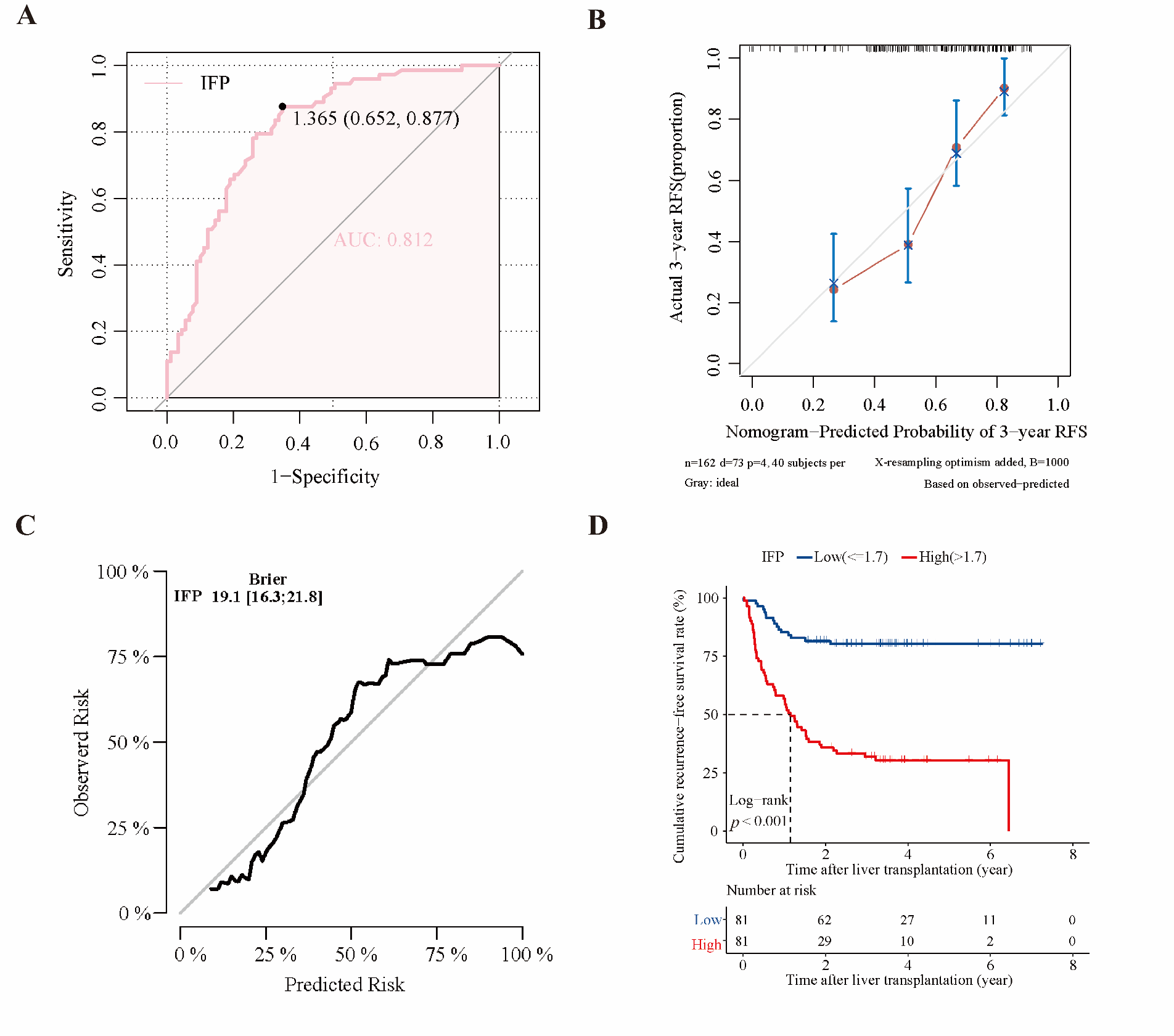


**Figure S7. The IFP predicts posttransplant HCC recurrence in the whole population after propensity score matching (n=162).** A. ROC curve for the IFP in the matched cohort. The AUROC was 0.812. B. Calibration curve of the IFP in predicting 3-year RFS in the matched cohort. C. The Brier score of the IFP in predicting 3-year RFS in the matched cohort (Brier score: 0.191, 95% CI, 0.163-0.218). D. RFS curve for the IFP in the matched cohort. Recipients in the low IFP and high IFP groups had significantly different RFS (*p* < 0.001). The 3-year RFS rate was 80.1% and 31.9%, respectively. PSM: propensity score matching; RFS: recurrence-free survival.


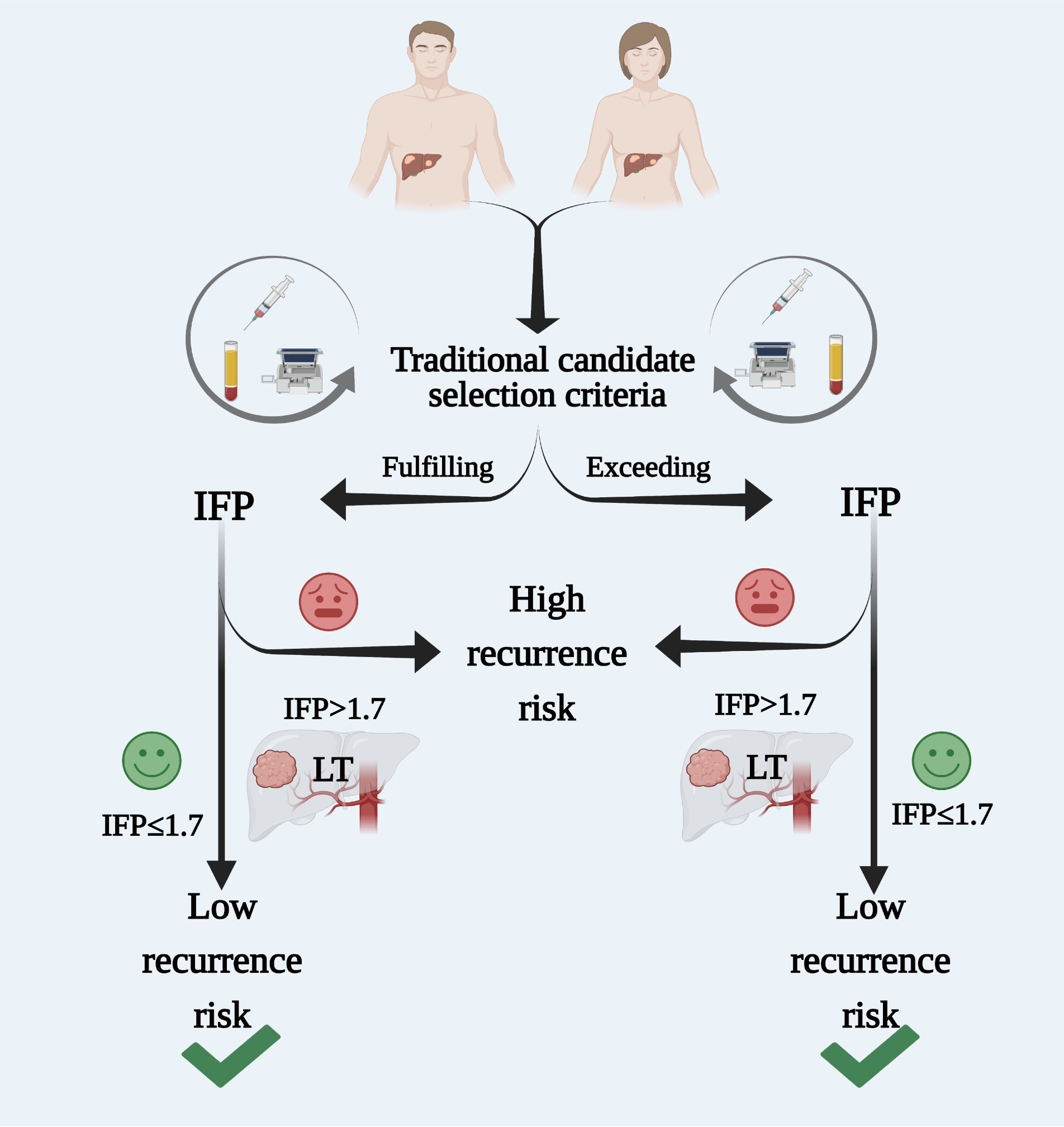


**Figure S8. The schematic diagram of the IFP in the clinical context.**
